# Are ChatGPT’s knowledge and interpretation ability comparable to those of medical students in Korea for taking a parasitology examination?: a descriptive study

**DOI:** 10.3352/jeehp.2023.20.1

**Published:** 2023-01-11

**Authors:** Sun Huh

**Affiliations:** Department of Parasitology and Institute of Medical Education, College of Medicine, Hallym University, Chuncheon, Korea; Hallym University, Korea

**Keywords:** Artificial intelligence, Educational measurement, Knowledge, Medical students, Republic of Korea

## Abstract

This study aimed to compare the knowledge and interpretation ability of ChatGPT, a language model of artificial general intelligence, with those of medical students in Korea by administering a parasitology examination to both ChatGPT and medical students. The examination consisted of 79 items and was administered to ChatGPT on January 1, 2023. The examination results were analyzed in terms of ChatGPT’s overall performance score, its correct answer rate by the items’ knowledge level, and the acceptability of its explanations of the items. ChatGPT’s performance was lower than that of the medical students, and ChatGPT’s correct answer rate was not related to the items’ knowledge level. However, there was a relationship between acceptable explanations and correct answers. In conclusion, ChatGPT’s knowledge and interpretation ability for this parasitology examination were not yet comparable to those of medical students in Korea.

## Graphical abstract

[Fig f2-jeehp-20-01]

## Background

O’Connor and ChatGPT [1] wrote an editorial, the opening paragraphs of which were written by ChatGPT, an artificial intelligence (AI) chatbot. ChatGTP was trained by a model using reinforcement learning from human feedback, using the same methods as InstructGPT (GPT: generative pre-trained transformer) [2]. AI chatbots such as ChatGPT could provide tutoring and homework help by answering questions and providing explanations to help students understand complex concepts. However, there are concerns that the use of AI software by students to write university assessments could diminish the value of the assessments and the overall quality of the university program [1]. After the release of ChatGPT to the public on November 30, 2022, it became a hot topic, particularly in education. Stokel-Walker [3] also noted that ChatGPT, an AI-powered chatbot that generates intelligent-sounding text in response to user prompts, including homework assignments and exam-style questions, has caused concern. Medical students must be able to evaluate the accuracy of medical information generated by AI and have the abilities to create reliable, validated information for patients and the public [4]. Therefore, it is necessary to determine how accurately ChatGPT, a recently developed AI chatbot, can solve questions on medical examinations. This comparison of ChatGPT’s abilities may provide insights into whether—and if so, how—medical students could use ChatGPT for their learning.

## Objectives

This study aimed to compare the knowledge and interpretation ability of ChatGPT with those of medical students in Korea by administering a parasitology examination. This subject is required in medical schools in Korea. Specifically, the following were investigated: (1) the scores of ChatGPT compared to those of the medical students; (2) the correct answer rate of ChatGPT according to items’ knowledge level; and (3) the acceptability of ChatGPT’s explanations as reflecting current parasitology knowledge, as evaluated by the author.

## Ethics statement

This was not a study of human subjects, but an analysis of the results of an educational examination routinely conducted at medical colleges. Therefore, neither receiving approval from the institutional review board nor obtaining informed consent was required.

## Study design

This is a descriptive study to compare the ability of ChatGPT to answer questions with that of medical students.

## Setting

On January 1, 2023 (Seoul time), a parasitology examination with identical items to those administered to first-year medical students at Hallym University on December 12, 2022, using computer-based testing (Supplement 1), was administered to ChatGPT (version December 15, 2022). The answers given by ChatGPT were compared to those of the medical students. Parasitology classes for medical students began on October 31, 2022, and ended on December 8, 2022. There were 16 hours of lectures and 32 hours of laboratory practice.

## Participants

Seventy-seven medical students took the parasitology on December 12, 2022. ChatGPT was counted as one examinee. There were no exclusion criteria.

## Variables

The items’ knowledge level and the examinees’ scores were the variables.

## Data sources and measurement

**Fig. 1.**
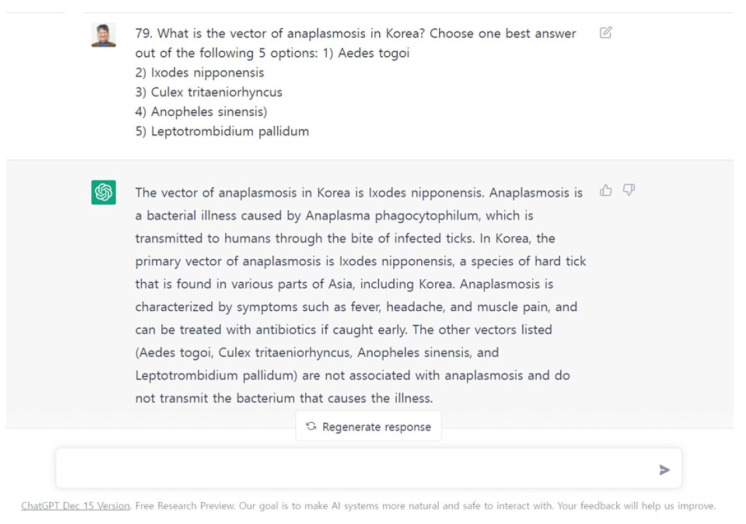
Screenshot of ChatGPT’s answer to a question item from a parasitology examination for medical students at Hallym University.

The response data of 77 medical students on the parasitology examination and ChatGPT were compared. The correct answer rate according to items’ level of knowledge was analyzed. The author also evaluated the acceptability of the explanations provided by ChatGPT (Supplement 2, Fig. 1), and classified the acceptability as good, needing revision, and unacceptable.

## Bias

There was no bias in the selection of examinees. All students who attended the parasitology lecture course were included.

## Study size

Sample size estimation was not required because all target students were included, and one AI platform was added.

## Statistical methods

Descriptive statistics were used to analyze the chatbot’s score. A comparative analysis was conducted using DBSTAT version 5.0 (DBSTAT).

## Score of ChatGPT and comparison with the medical students’ performance

According to data from Dataset 1, ChatGPT correctly answered 48 out of 79 items (60.8%). This score was lower than the average score of 77 medical students, which was 71.8 out of 79 (90.8%), with a minimum score of 65 (89.0%) and a maximum score of 74 (93.7%).

## Comparison of ChatGPT’s correct answer rate according to items’ knowledge level

Table 1 shows ChatGPT’s responses according to items’ knowledge level. The chi-square test yielded results of *χ*^2^=3.02, degrees of freedom (df)=2, with a significance level of 0.05 (*χ*^2^=5.99). This result indicates that the relationship between the 2 variables was not significant (P=0.2206).

## Acceptability of ChatGPT’s explanations

Table 2 shows the acceptability of ChatGPT’s explanations according to the correctness of the answer. The chi-square test showed results of *χ*^2^=51.62, df=2, with a significance level of 0.05 (*χ*^2^=5.99). This result indicates that the relationship between the 2 variables was significant (P=0.0000).

## Key results

ChatGPT’s performance was lower than that of medical students. The correct answer rate shown by ChatGPT was not related to the items’ knowledge level. However, there was an association between acceptable explanations and correct answers.

## Interpretation

ChatGPT’s correct answer rate of 60.8% was not necessarily an indicator of poor performance, as the questions were not easy for medical students to answer correctly. The considerably higher average score (89.6%) of the medical students may have been due to their prior learning of parasitology and the fact that the examination was administered 4 days after the class. If the examination had been taken 1 or 2 months after the class, the students’ performance scores might have been lower. Some incorrect answers may have been due to the following factors: first, ChatGPT is currently unable to interpret figures, graphs, and tables as a student can, so the author had to describe these materials in text form. Second, some epidemiological data unique to Korea were outside ChatGPT’s knowledge. Some of those data are only available in Korean or are not searchable online. Third, ChatGPT sometimes did not understand multiple-choice questions where the examinee must select the best answer out of multiple options. ChatGPT sometimes selected 2 or more options, as it has not yet been trained to do otherwise.

There was no significant difference in the correct answer rate according to the knowledge level of the items. However, this may vary in other examinations and may have been a unique phenomenon for this parasitology exam. ChatGPT’s explanations of the question items were generally acceptable if it made a correct selection. However, the explanations for 7 items needed to be updated or revised because they contained incorrect information. This finding suggests that ChatGPT’s knowledge in specific fields (e.g., parasitology) remains insufficient. If the incorrect option was selected, the explanation was unacceptable or needed revision in 90.0% of items. This result was anticipated, as students’ explanations for incorrect selections are also usually unacceptable. Sometimes, GPT could not select best answer but, the explanation is acceptable. Example is the item number 39.

## Comparison with previous studies

There have been no reported studies in the literature databases, including PubMed, Scopus, and Web of Science, on the comparability of ChatGPT’s performance to that of students on medical examinations.

## Limitations

The input for the question items for ChatGPT was not precisely the same as for the medical students. The chatbot cannot receive information in graphs, figures, and tables, so this information was re-described by the author. Additionally, the interpretation of the explanations and correct answers may vary according to the perspectives of different parasitologists, although the author has worked in the field of parasitology for 40 years (1982–2022) in Korea. Best practices for patient care may also vary according to the region and medical environment.

## Generalizability

The above results cannot be generalized directly to other subjects or medical schools, as chatbots will likely continue to evolve rapidly through user feedback. A future trial with the same items may yield different results. The present results reflect the abilities of ChatGPT on January 1, 2023.

## Implications for medical/health students and professors to use ChatGPT

Currently, ChatGPT’s level of knowledge and interpretation is not sufficient to be used by medical students, especially in medical school exams. This may also be the case for high-stakes exams, including health licensing exams. However, I believe that ChatGPT’s knowledge and interpretation abilities will improve rapidly through deep learning, similar to AlphaGo’s ability [5]. Therefore, medical/health professors and students should be mindful of how to incorporate this AI platform into medical/health education soon. Furthermore, AI should be integrated into the medical school curriculum, and some schools have already adopted it [6].

## Conclusion

ChatGPT’s knowledge and interpretation ability in answering this parasitology examination are not yet comparable to those of medical students in Korea. However, these abilities will likely improve through deep learning. Medical/health professors and students should be aware of the progress of this AI chatbot and consider its potential adoption in learning and education.

## Figures and Tables

**Figure f2-jeehp-20-01:**
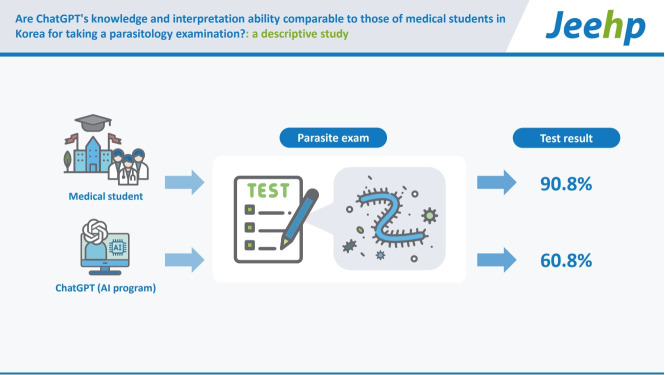


**Table 1.** Correct responses by ChatGPT according to the knowledge level of 79 items

Knowledge level of items	Correct responses	Incorrect answers
Recall	17	15
Interpretation	20	12
Problem-solving	11	4

**Table 2.** Acceptability of ChatGPT’s explanations of the 79 question items by correctness of the answer

Explanation	Correct answers	Incorrect answers
Good	41	3
Needs to be revised	7	8
Unacceptable	0	20

## Footnotes

**Data availability**
Data files are available from Harvard Dataverse: https://doi.org/10.7910/DVN/N7NKSQ Dataset 1. Raw data for analysis, including item number, knowledge level, correct answer, ChatGPT’s answers, and correctness of explanations for a parasitology examination taken by the first-year medical students at Hallym University on December 12, 2022..

## Supplementary materials

Supplementary files are available from Harvard Dataverse: https://doi.org/10.7910/DVN/N7NKSQ

Supplement 1. Seventy-nine items from a parasitology examination taken by first-year medical students at Hallym University on December 12, 2022.

Supplement 2. ChatGPT’s responses to 79 items from a parasitology examination taken by first-year medical students at Hallym University on December 12, 2022, inputted on January 1, 2023, by the author. Figures and tables are removed and explained in the item stem. Explanations of the options selected by ChatGPT are also included.

Supplement 3. Audio recording of the abstract.

## References

[1] O’Connor S (2022). Open artificial intelligence platforms in nursing education: tools for academic progress or abuse?. Nurse Educ Pract.

[2] OpenAI (2022). ChatGPT Dec 15 version [Internet]. https://chat.openai.com/chat.

[3] Stokel-Walker C (2022). AI bot ChatGPT writes smart essays: should professors worry?. Nature.

[4] Park SH, Do KH, Kim S, Park JH, Lim YS (2019). What should medical students know about artificial intelligence in medicine?. J Educ Eval Health Prof.

[5] Silver D, Huang A, Maddison CJ, Guez A, Sifre L, van den Driessche G, Schrittwieser J, Antonoglou I, Panneershelvam V, Lanctot M, Dieleman S, Grewe D, Nham J, Kalchbrenner N, Sutskever I, Lillicrap T, Leach M, Kavukcuoglu K, Graepel T, Hassabis D (2016). Mastering the game of Go with deep neural networks and tree search. Nature.

[6] Hu R, Fan KY, Pandey P, Hu Z, Yau O, Teng M, Wang P, Li A, Ashraf M, Singla R (2022). Insights from teaching artificial intelligence to medical students in Canada. Commun Med (Lond).

