# Supplementary material for: Are ChatGPT’s knowledge and interpretation ability comparable to those of medical students in Korea for taking a parasitology examination?: a descriptive study
Source: J Educ Eval Health Prof. 2023 Jan 11;20:1. doi: 10.3352/jeehp.2023.20.1 (PMC9905868; doi:10.3352/jeehp.2023.20.1)
Supplement: Supplementary file 2 — Supplement 1. Seventy-nine items from a parasitology examination taken by first-year medical students at Hallym University on December 12, 2022. [file jeehp-20-1-suppl1.pdf]

CBT Parasitology- Dec 12, 2022, Hallym University -79 items

CASE> A 45-year-old female patient visited a nearby hospital for abdominal discomfort that occurred two weeks before her visit to the hospital. She was diagnosed with urinary tract infection, and was treated with antibiotics. After treatment, she complained of minor gastrointestinal symptoms continuously. Gastric endoscopy was performed but no special findings were observed in the upper gastrointestinal endoscopy. The colonoscopy was done also. Result of colon mucous membrane surface biopsy were shown in the figure. Parasitic pathogen can be seen the black circle marking.

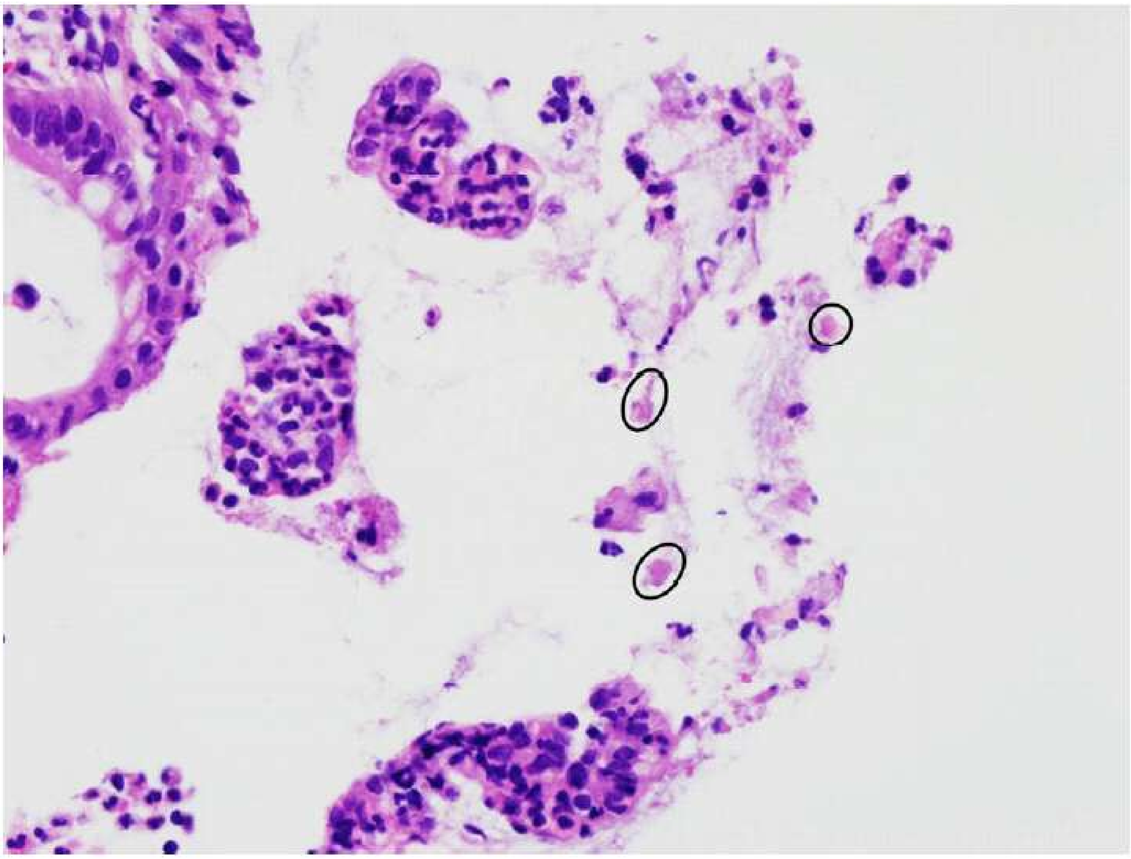

Figure. Result of colon mucous membrane surface biopsy from 45 year-old female patient who complains of abdominal discomfort.

1. What is the scientific name of this parasite?

1) *Entamoeba histolytica*
2) *Entamoeba coli*
3) *Giardia lamblia*
4) *Endolixmax nana*
5) *Trichomonas vaginalis*

답) 원충, 이질아메바, 판단형 1

2. What is the therapeutic drug for this patient?

1) Albendazole
2) Praziquantel
3) Primaquine
4) Metronidazole
5) Ivermectin

답) 원충, 이질아메바, 문제해결형 4

3. What is the history for cause of infection?

1) Eating raw pork
2) Eating raw fresh water fish
3) Eating raw beef
4) Eating vegetable during the travel to Vietnam
5) Eating raw raw oyster

답) 원충, 이질아메바, 암기형 4

4. What is the mechanism of causing lesion in the intestine?

1) Intrusion to crypt
2) Covering villous membrane
3) Invasion to mucosa
4) Allergic reaction to the excretory protein of the parasite
5) Increased number of the secretory gland

답) 원충, 이질아메바, 암기형 3

Case> A 45-year-old man who complained of the abdominal pain and diarrhea visited an outpatient clinic. Figure was observed by stool stain. The diameter of the parasite was 5 micrometers.

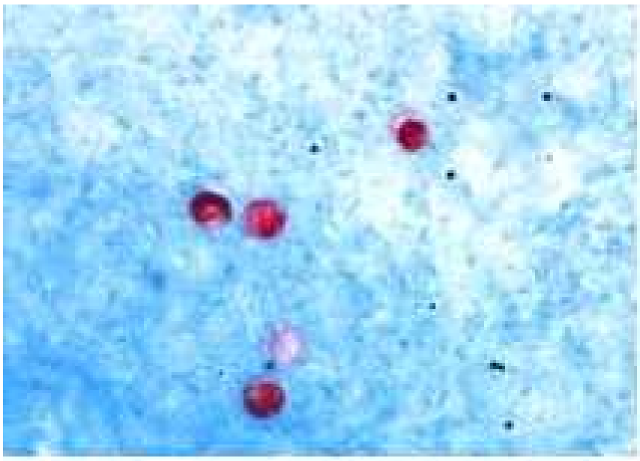

5. What is the name of causative parasite?

1) *Microsporidia* spp.
2) *Cyclospora* spp.
3) *Cryptosporidium* spp.
4) *Isospora* spp.
5) *Giardia* spp.

답> 원충, 작은와포자충, 판단형 3

6. What is the stain method of stool used in this case?

1) Modified acid-fast stain
2) Giemsa stain
3) Iron-hematoxylin stain
4) Hmatoxylin-eosin stain
5) Trichrome stain

답> 원충, 작은와포자충, 암기형 1

7. What is the appropriate treatment method?

1) Prescription of praziquantel
2) Prescription of albendazole
3) Prescription of chloroquine

4) Prescription of metronidazole
5) Conservative measure

A> 원충, 작은와포자충, 문제해결형 5

8. What is the highly specific diaganostic methods besides of stool exam?

1) Indirect immuno-fluorescent assay
2) Polymerase chain reaction
3) Enzyme linked immunosorbent assay
4) Western blot
5) Northern blot

A> 원충, 작은와포자충, 문제해결형 2

9. What is the source of infection in this patient?

1) Eating raw pork
2) Eating raw fresh water fish
3) Eating raw beef
4) Eating vegetable
5) Eating raw raw oyster

답> 원충, 작은와포자충, 암기형 4

증례> From May 2013 to November 2013, multiplex polymerase chain reaction was performed for male patients visiting a urologist in Daegu. The results are shown in Tables 1 and 2.

**Factor**	**Characteristic**	**No. of patients**	***T. vaginalis* prevalence (%)**
Overall		201	8 (4.0)
Age (years)	10-19	2	0 (0.0)
	20-29	9	1 (11.1)
	30-39	26	0 (0.0)
	40-49	35	2 (5.7)
	50-59	53	3 (5.7)
	60-69	48	1 (2.1)
	70-79	26	1 (3.8)

	80-89	2	0 (0.0)
Marital status	Single	28	1 (3.6)
	Married	150	6 (4.0)
	Divorced	4	1 (25.0)
	Missing	19	0 (0.0)
Urogenital symptoms	None	75	3 (4.0)
	Penile discharge	11	0 (0.0)
	Penile tingling	28	1 (3.6)
	Pain during urination	98	4 (4.1)
	Lower abdominal pain	16	0 (0.0)

Table 1. Analysis of *Trichomonas vaginalis* infection status according to sociodemographic characteristics and urogenital symptoms (N=201)

No.	Age	PCR		Multiplex PCR						Disease
		**Tvk**	**BTUB**	**TV**	**MH**	**MG**	**CT**	**NG**	**US**	
**1**	***43***	**+**	**+**	**-**	**-**	**-**	**-**	**-**	**-**	**Prostatitis**
**2**	***28***	**+**	**+**	**-**	**-**	**-**	**+**	**-**	**-**	**Prostatitis**
**3**	***74***	**+**	**+**	**-**	**-**	**-**	**+**	**-**	**-**	**BPH**
**4**	***52***	**+**	**+**	**-**	**-**	**-**	**-**	**-**	**-**	**Prostatitis**
**5**	***56***	**+**	**+**	**-**	**-**	**-**	**-**	**-**	**-**	**Prostatitis**
**6**	***53***	**+**	**+**	**-**	**-**	**+**	**-**	**-**	**-**	**Urethritis**
**7**	***46***	**+**	**+**	**-**	**-**	**-**	**-**	**-**	**-**	**BPH**
**8**	***67***	**+**	**+**	**-**	**-**	**-**	**-**	**-**	**-**	**BPH**

Table 2. Detection of sexually transmitted infection microorganisms in *Trichomonas*-positive 8 patients

TV, *Trichomonas vaginalis*; MH, *Mycoplasma hominis*; MG, *Mycoplasma genitalium*; CT, *Chlamydia trachomatis*; NG, *Neisseria gonorrhoeae*; US, *Ureaplasma* sp.; BPH, *benign prostatic hyperplasia*; BTUB, β-tubulin gene from *T. vaginalis*.

10. What are the most common symptoms of men's trichominasis according to Tables?

1) Penile discharge

2) Penile tingling
3) Pain during urination
4) Lower abdominal pain
5) Frequent urination

A> 원충, 질편모충, 판단형 3

11. What is the therapeutic drug for above *Trichomonas* positive visitors?

1) Albendazole
2) Praziquantel
3) Primaquine
4) Metronidazole
5) Ivermectin

A> 원충, 질편모충, 문제해결형 4

12. What is the mechanism causing lesion in the vagina of T*richomonas vaginalis* infected female?

1) Harboring in the vaginal epithelium
2) Covering vaginal epithelium so that increasing secretion
3) Changing the vaginal pH into acidic status
4) Inhibiting the proliferation of the vaginal epithelium
5) Inducing chemolucence of neutrophil by the excretory-secretory protein

A> 원충, 질편모충, 암기형 5

증례> A 37-year-old man presented with a 3-month history of cutaneous lesions on the left postauricular area and left arm. The patient had experienced several insect bites while traveling to the Amazon region in Brazil five months ago. On physical examination, Fig. 1 was found. Skin biopsies of the lesions were done and histopathological finding was as Fig. 2.

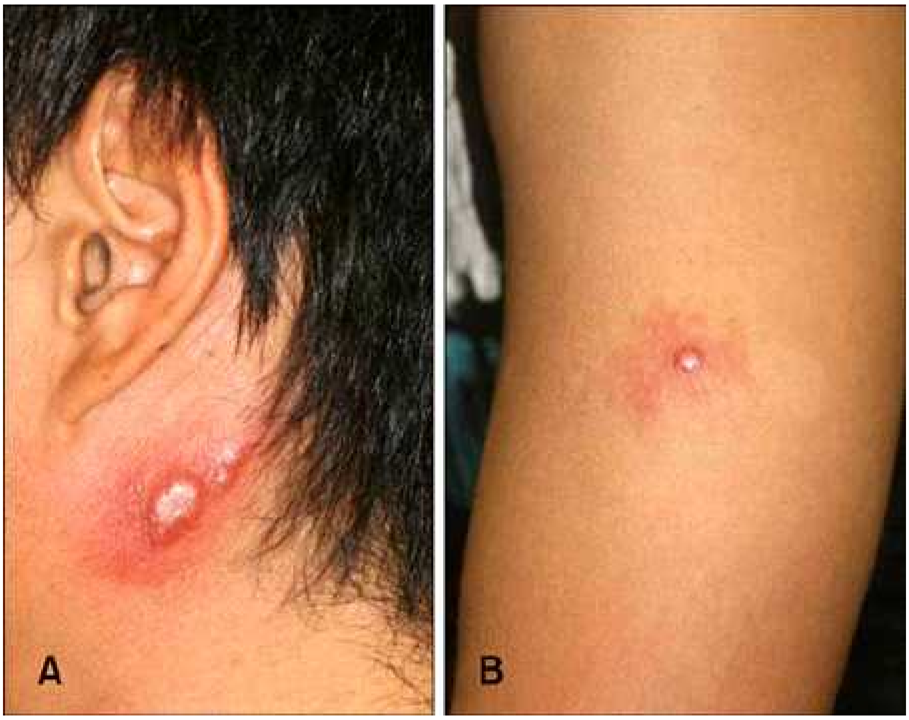

Fig. 1

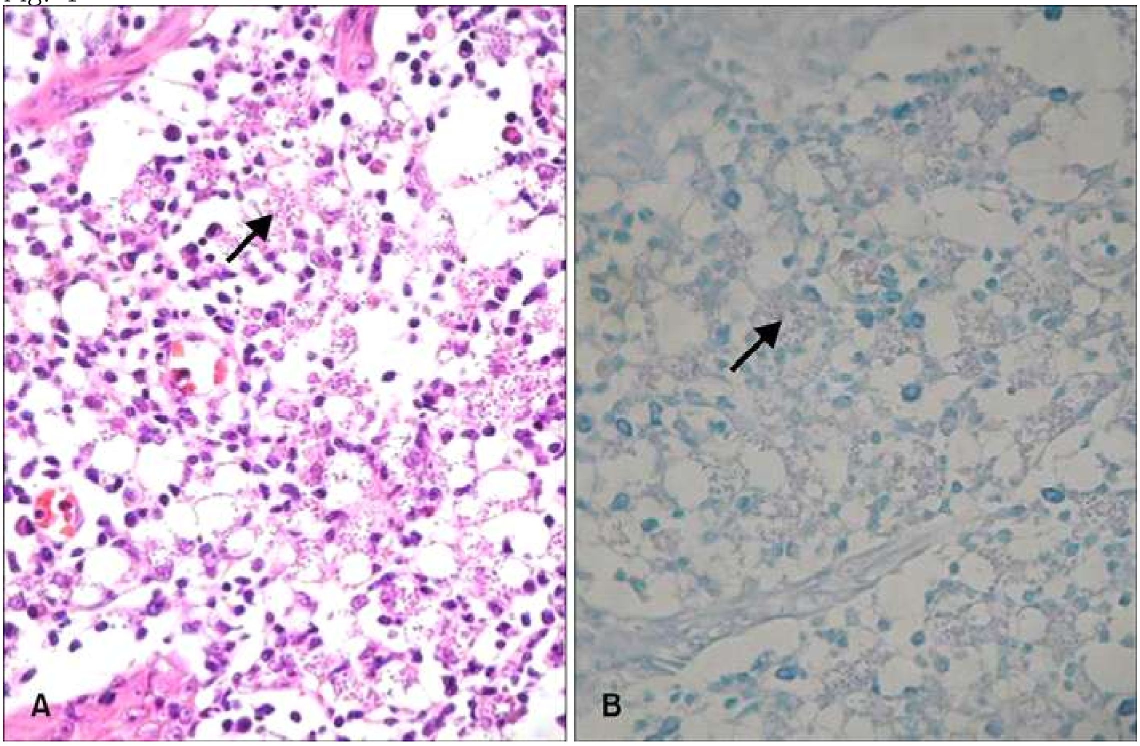

Fig. 2.

13. What is the causative agent of this disease?

1) *Leishmania braziliensis*
2) *Leishmania donovani*
3) *Trypanosoma brucei gambiense*
4) *Trypanosoma brucei rhodesiense*
5) *Trypanosoma cruzi*

A> 원충, 피부리슈만편모충증, 판단형 1

14. What is the drug for treatment?

1) sodium stibogluconate injection to the lesion
2) Praziquantel
3) Primaquine
4) Metronidazole
5) Ivermectin

A> 원충, 피부리슈만편모충증, 문제해결형 1

15. What is the vector of this disease?

1) sand fly
2) tsetse fly
3) kissing bug
4) mite
5) mosquito

A> 원충, 피부리슈만편모충증, 암기형 1

증례> In March 2003, 13 adults went hunting in Inje, Gangwon Province, and ate wild boars and badgers captured. They complained of high fever, muscle pain, and facial edema. Tissue biopsy findings are illustrated in the figure.

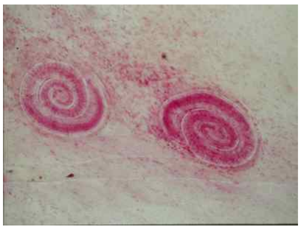

Figure. Biopsy finding of patient's muscle.

16. What is the causative agent?

1) *Trichinella spiralis*
2) *Gnathostoma spinigerum*
3) *Anisakis simplex* larvae
4) *Toxocara canis* larvae
5) *Brugia malayi*

답> 선충, 선모충, 판단형 1

17.What is the therapeutic drug to patients?
1) Levamisole
2) Ivermectin
3) Praziquantel
4) Diethyl carbamazine
5) Albendazole

답> 선충, 선모충,문제 해결형 5

증례> Walking barefoot along the coast resulted in the following findings.

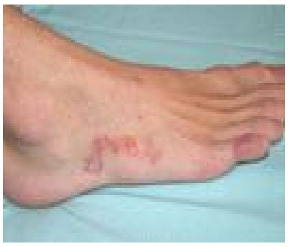

18. What is the causative agent?

1) *Ancylostoma duodenale*
2) *Ascaris lumbricoides*
3) *Trichuris trichiura*
4) *Oncocerca volvulus*
5) *Brugia malayi*

답> 선충, 두비니구충, 판단형 1

Case> A 38-year-old man visited an outpatient clinic in Gabon, Africa, with the following findings.

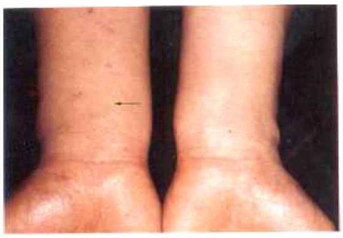

Fig. 1. Diffuse swelling of the right hand and arm

19. What is the causative agent?

1) Wucheria bancrofti
2) Bruia malayi
3) Onchocerca volvulus
4) Loa loa
5) Dirofilaria immitis

답>선충, 로아사상충, 판단형 4

Case> Figure is the photo of the parasite.

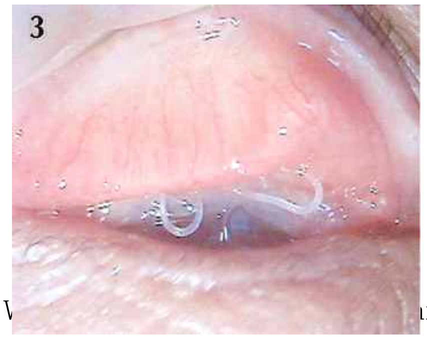

20. W[illegible]nd its developmental stage?

1) *Trichinella spiralis* larva

2) *Toxocara canis* larva
3) *Thelazia callipaeda* adult
4) *Brugia malayi* filaria
5) *Anisakis simplex* larva

답> 선충, 동양안충, 판단형, 3

Case> A 72-day-old girl died after being hospitalized in an intensive care unit due to breathing difficulties. Figure 1 shows the result of observing Leukostat stain by smear printing the autopsy lung tissue. Figure 2 shows the findings of the postmortem lung.

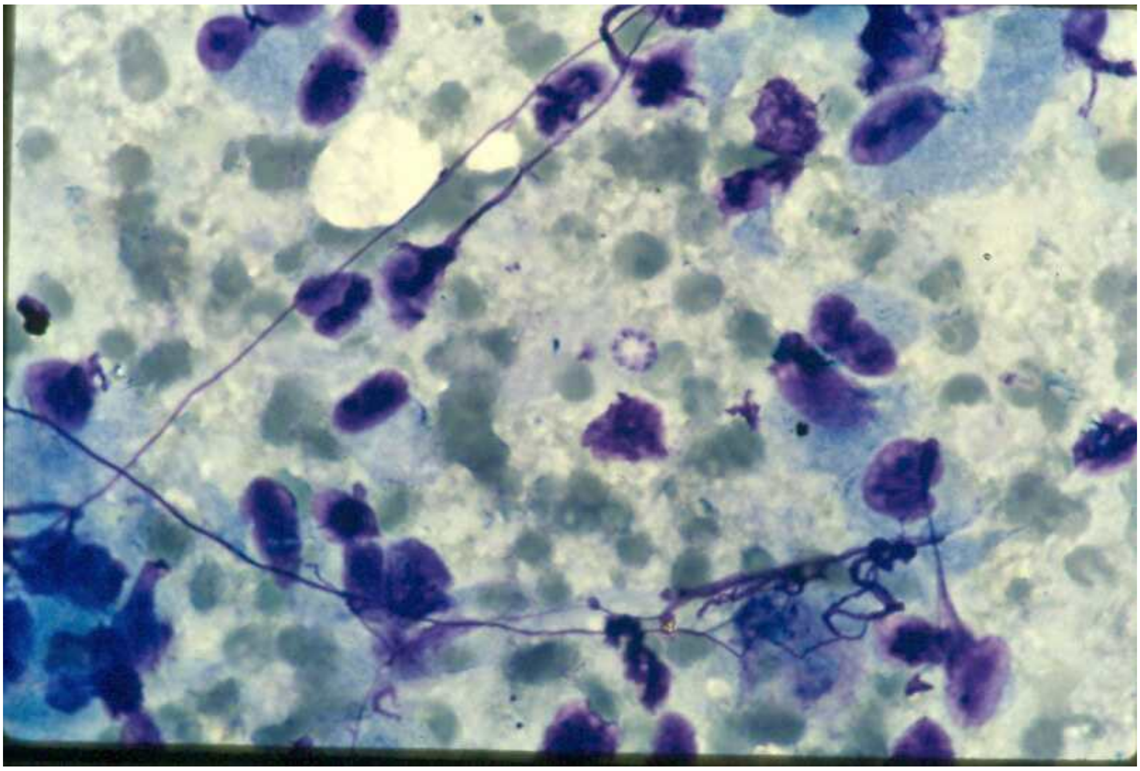

Figure 1. Lung tissue print smear with Leukostat stain.

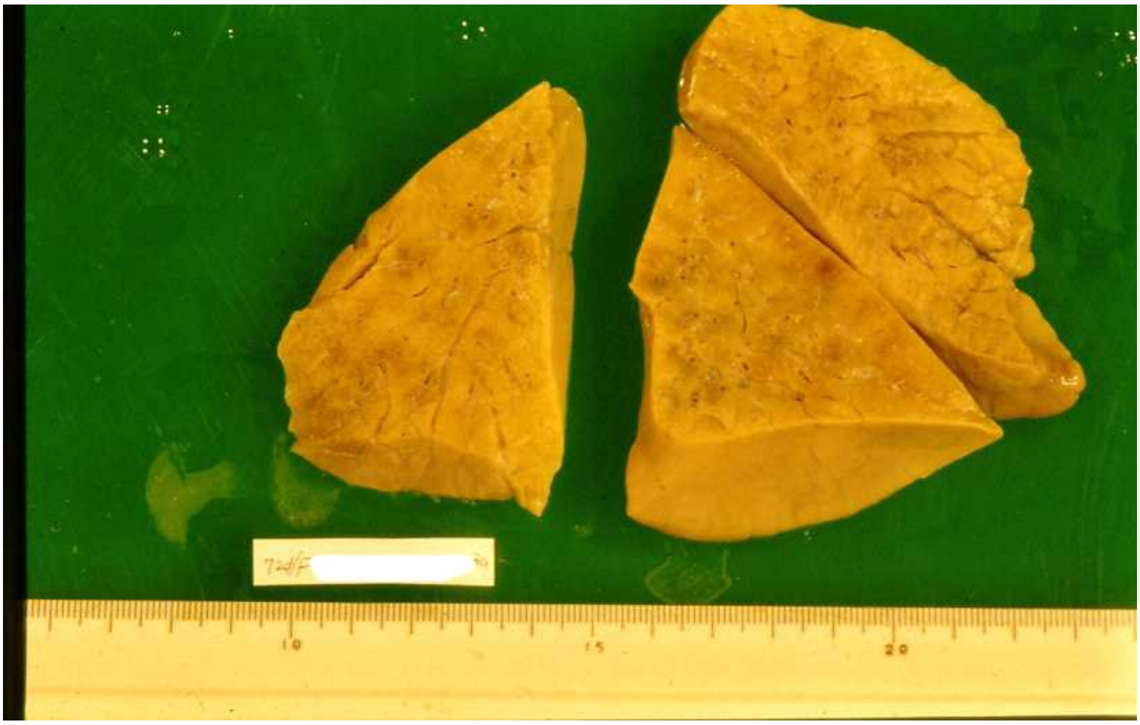

Figure 2. The postmortem lung of 72 days-old child who suffered from breathing difficulties.

21. What is the causative agent?

1. *Cryptosporidium parvum*
2. *Toxoplasm gonddii*
3. *Giardia lamblia*
4. *Pneumocystis carinii*
5. *Naegleria fowleri*

답) 진균, 폐포자충, 판단형 4

22. What is the drug of choice for this infection?

1. Trimethoprim / sulfamethoxazole
2. Metronidazole
3. Albendazole
4. Praziquantel
5. Primaquine

답) 진균, 폐포자충, 문제해결형 1

Case> A 7-year-old child had a torn left eyebrow, and after being sutured, a kidney-sized nodule developed in the wound and was treated with antibiotics, fungicides and steroids for 42 days. In the skin biopsy, the trophozoite as in the Figure was observed. While the wound was progressing, the child was hospitalized due to vomiting, lack of appetite, and a mild fever. The cerebrospinal fluid was clear, with negative gram-stain, bacterial culture, and Indian ink. On the 10th day of hospitalization, brain CT imaging showed the dense density in the left temporal lobe. The child became unconscious and died within a day later.

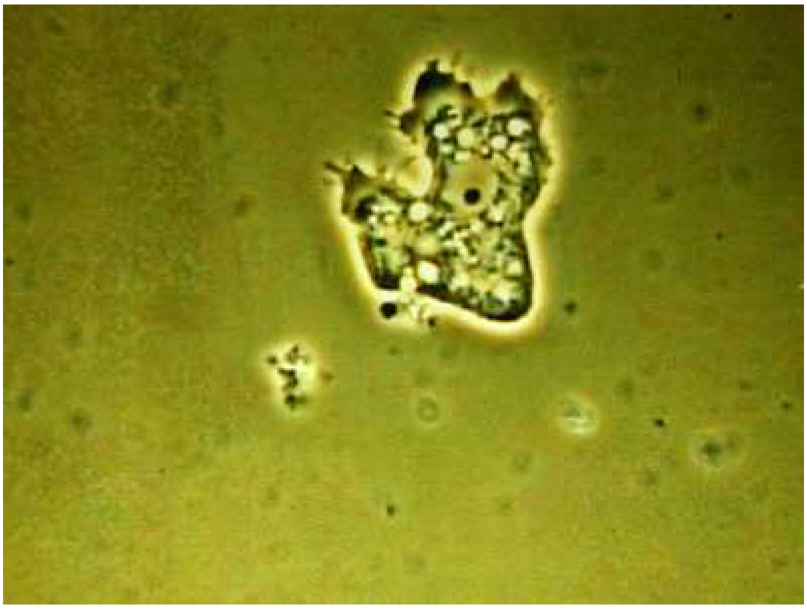

Figure. Trophozoites from skin biopsy

23. What is the causative agent?

1. *Cryptosporidium parvum*
2. *Toxoplasm gonddii*
3. *Giardia lamblia*
4. *Pneumocystis carinii*
5. *Acanthamoeba culbertsoni*

답> 원충, 자유생활아메바, 판단형 5

case> A 68-year-old woman residing in Busan, Korea, had epigastric pain with severe vomiting about 5 hours after eating a) foods. Worms penetrating the gastric mucosae in the great curvature of the middle body and fundus were found and

removed during gasteroendoscopic examination as Figures.

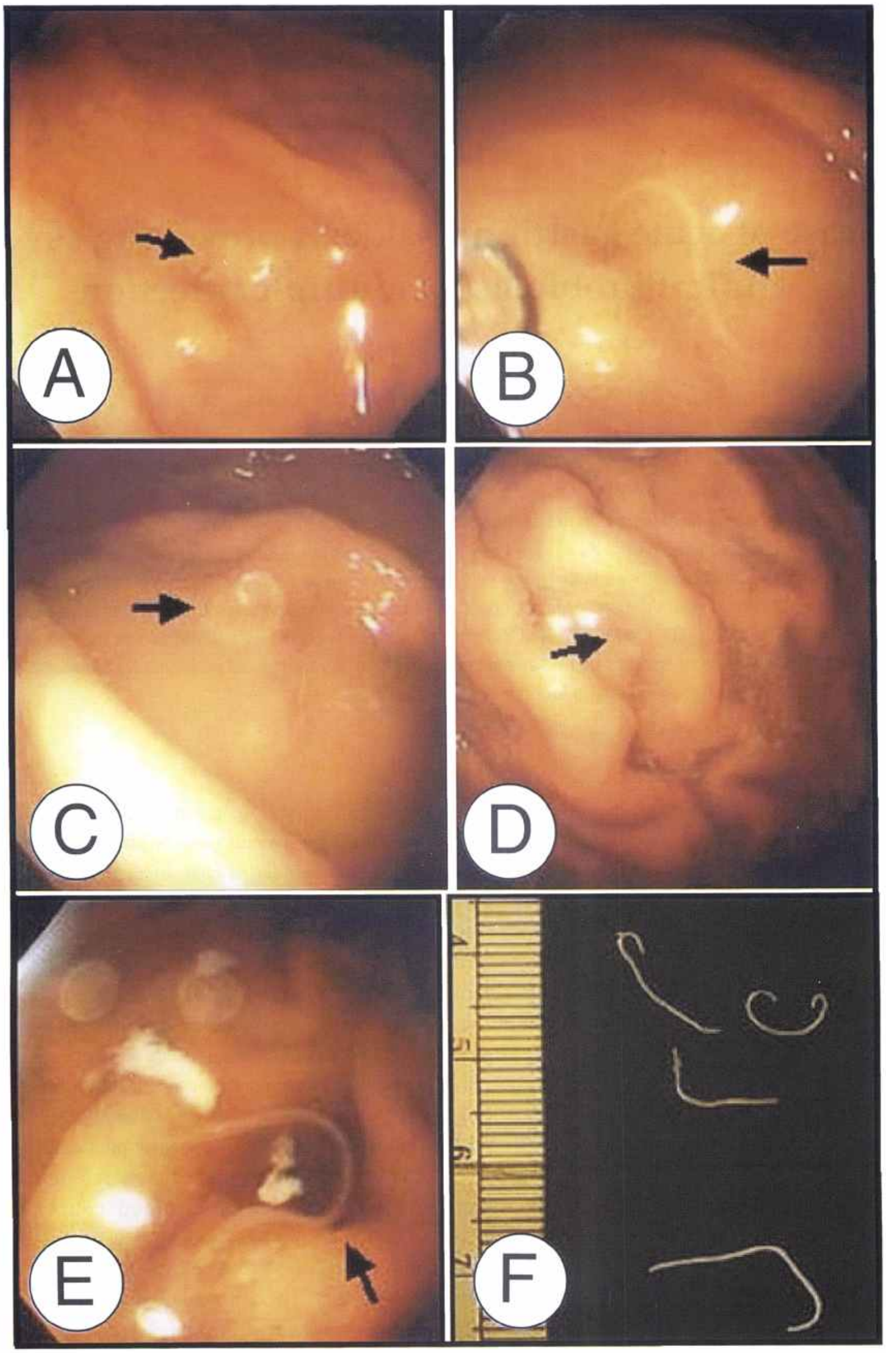

Figures. Endoscopic finding of patient who had epigastric pain with severe vomiting

24. What is the causative pathogen?

1) Larvae of *Spirometra erinacei*
2) Larvae of *Taenia solium*
3) Larvae of *Toxocara canis*
4) Larvae of *Anisakis simplex*
5) Larvae of *Ascaris lumbricoides*

답>   선충, 고래회충 판단형 4

25. What is the a)foods suspected?

1) raw beef
2) raw anchovy
3) raw snake
4) vegetable
5) raw cow liver

답> 선충, 고래회충, 암기형 2

26. Which is the correct description of the scientific name of the parasite?

1) *Toxocara Canis*
2) *To. canis*
3) *TOXOCARA CANIS*
4) *T. Canis*
5) *T. c.*

답> 총론, 학명, 암기형 3

27. What is the mode of transmission of *Enterobius vermicluaris*?

1) snail-transmitted
2) soil-transmitted
3) arthropod-borne
4) contact-borne
5) food-borne

답> 선충, 요충, 암기형 4

28. What is the mode of transmission of *Ascaris lumbricoides*?

1) snail-transmitted
2) soil-transmitted
3) arthropod-borne
4) contact-borne
5) food-borne

답> 선충, 회충, 암기형. 2

29. “A life style in which one species organism temporarily or permanently inhabits in the body or surface of another species organism, taking nourishment“ is called

1) parasitism
2) commensalism
3) mutualism
4) symbiosis
5) free living
답> 총론, 용어 정의, 암기형 1

30. ”An animal (or species) that is infected by a parasite, and which serves as a

source of infection for humans or another species“ is called

1) intermediate host
2) vector
3) final host
4) reservoir host
5) paratenic host

답> 총론, 용어 정의, 암기형 4

31. “Blood-sucking arthropods that spread parasites from one host to another, serving as intermediate host or final host“ is called

1) intermediate host
2) vector
3) final host
4) reservoir host
5) paratenic host

답> 총론, 용어 정의, 암기형 2

32. The following is a graph the change of egg positive rate of a parasite from 1972 to 2012. What is the name of this parasite?

1) Ascaris lumbricoides
2) Trichuris trichiura
3) Enterobius vermicularis
4) Ancylostoma duodenale
5) Toxocara canis

답> 선충, 요충, 판단형 3

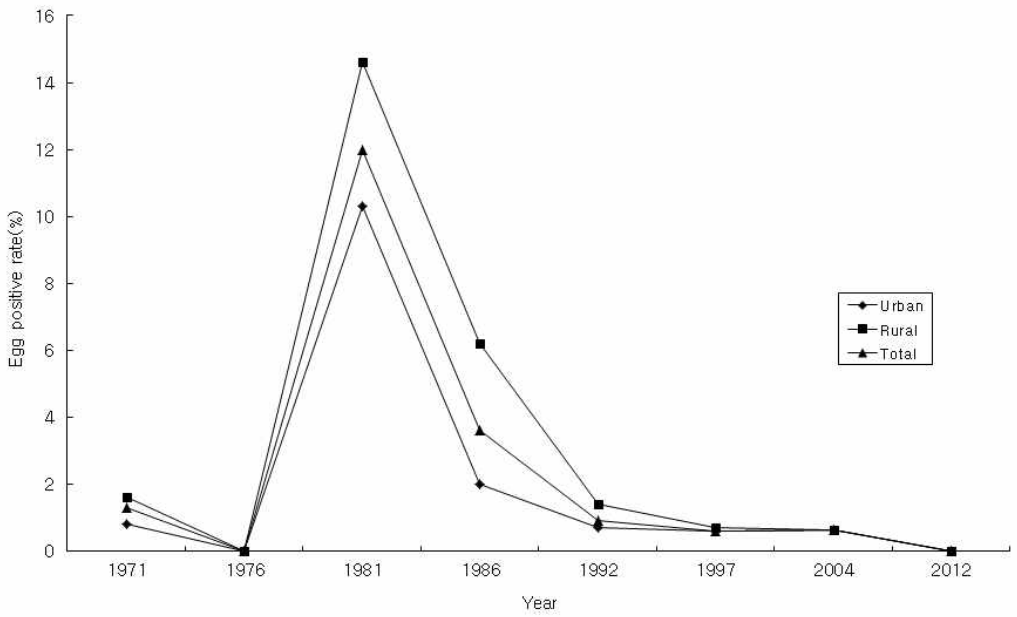

조사년도별, 도시/농촌별 충란 양성률

Case> Following is the parasite collected from bile duct of the 56 year-old man who were operated due to bile duct occlusion.

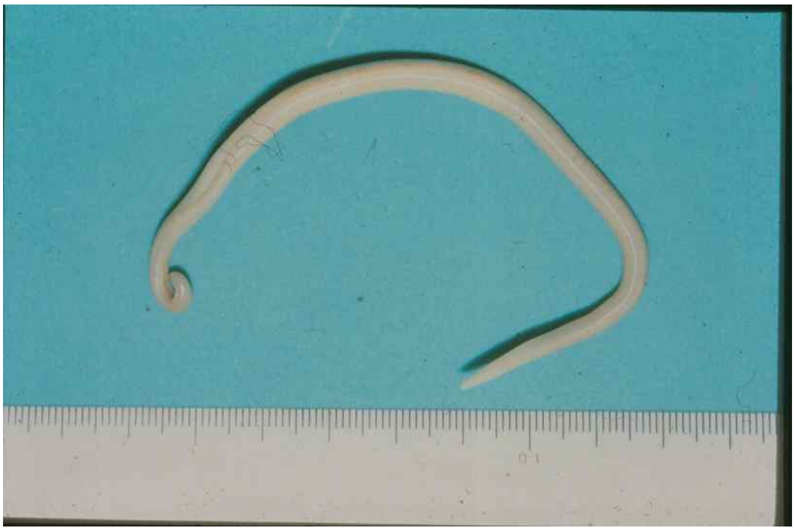

33. What is the name of parasite?

1) Ascaris lumbricoides
2) Enterobius vermicularis
3) Trichuris trichiura
4) Brugia malayi
5) Anisaksi simplex

A> 선충, 회충, 판단형, 1

Case> During colonoscoy, worm was found in the cecum (A): Worm recovered was stained with H-E stain (B); Eggs of worm was found (C); Histopathologic founding of lamina propria was shown (D) in Figure.

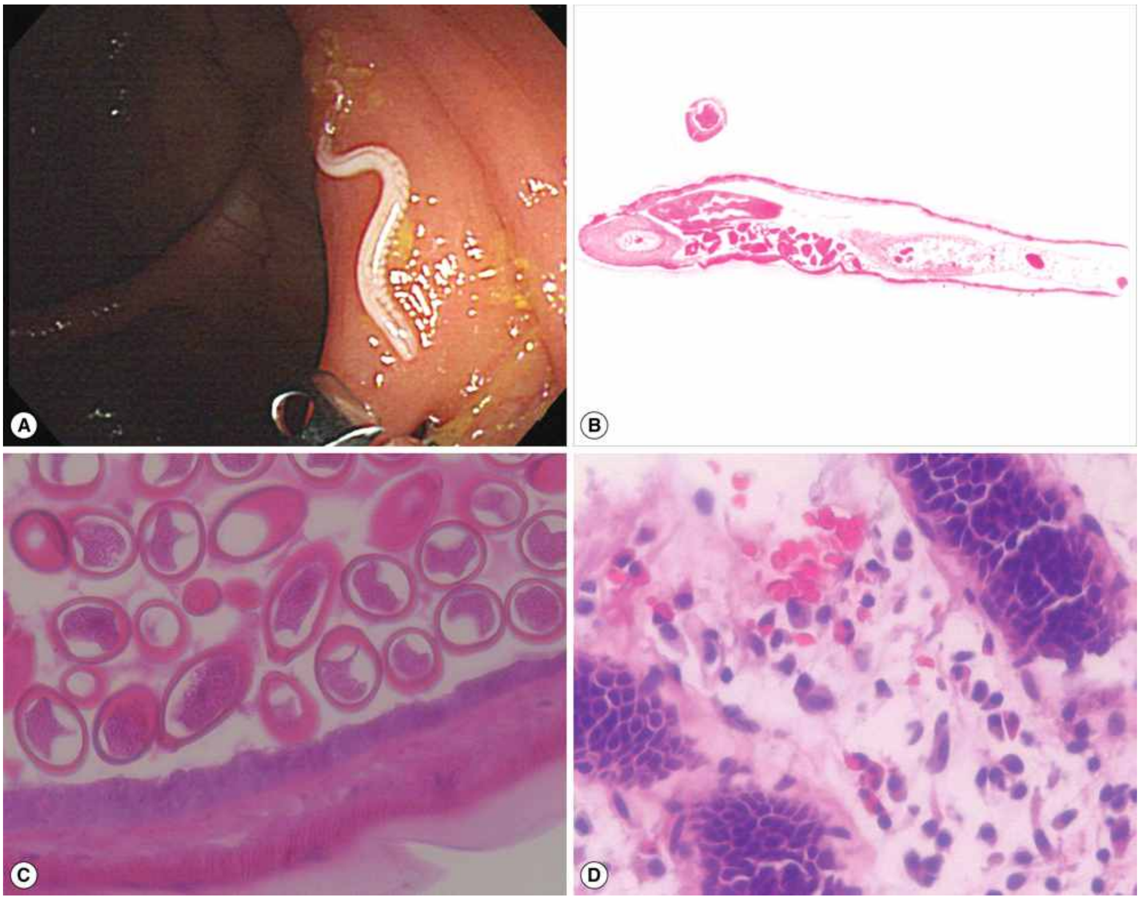

Fig. Colonoscopic finding and microscopic finding of worm.

34. What is the worm recovered?

1) *Trichuris trichiura*
2) *Ascaris lumbricoides*
3) *Enterobius vermicularis*
4) *Ancylostoma duodenale*
5) *Capillaria philippinensis*

A> 선충, 편충, 판단형 1

35. What is the therapeutic drug to this parasite?

1) Metronidazole
2) Albendazole
3) Praziquantel
4) Chloroquine
5) Crotamiton

a> 선충, 편충, 문제해결형 2

36. What is the mode of transmission of this worm?

1) Snail-transmitted
2) Soil-transmitted
3) Contact-borne
4) Food-borne
5) Vector-borne

a> 선충, 편충, 암기형 2

Case> A 4 year-old girl visited a health center. Her mother said that she complains of itching of anus at night. Following eggs were found from her. Egg length was 60 micrometer.

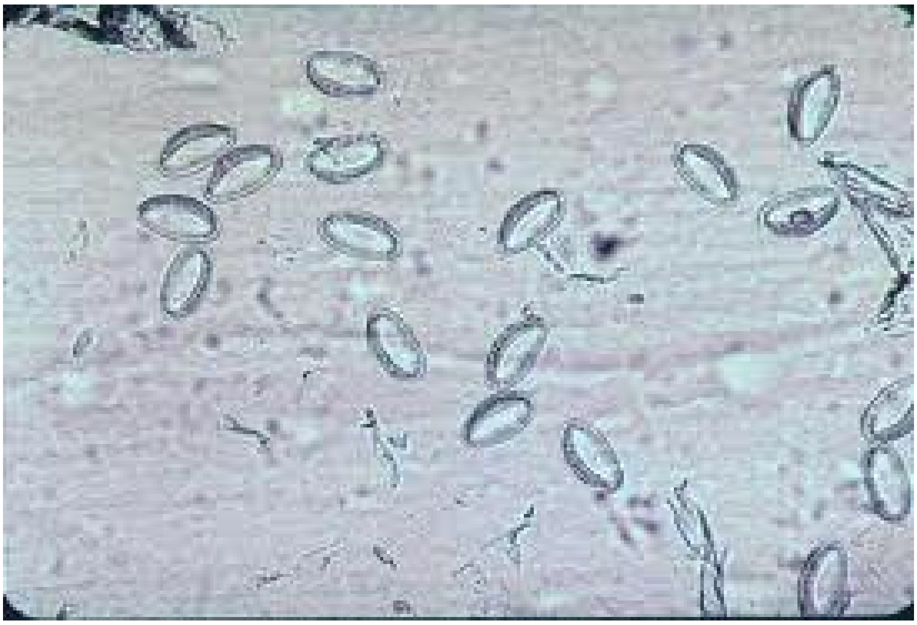

Fig. Microscopic finding.

37. What is the diagnosis method of this girl's problem?

1) Stool examination
2) Perianal swab
3) Serum ELISA
4) PCR
5) Colonoscopy

답> 선충, 요충, 암기형, 2

38. What is the causative pathogen in this case?

1) *Trichuris trichiura*
2) *Enterobius vermicularis*
3) *Ascaris lumbricoides*
4) *Ancylostoma duodenale*
5) *Necator americanus*

a> 선충, 요충, 판단형, 2

39. What is the therapeutic drug?

1) Metronidazole
2) Albendazole
3) Praziquantel
4) Chloroquine
5) Crotamiton

a> 선충, 요충, 문제해결형 2

40. What is the best method of treatment mode for this girl?

1) Three times medication repeated every three weeks
2) Two times medication repeated every two weeks
3) Two times medication repeated every one week
4) One day medication, twice a day
5) One time medication

a> 선충, 요충, 문제해결형 1

Case> A 45-year-old-male who had underlying ulcerative colitis and presented with fever and dry cough. Following is the chest X-rays showing consolidations in the right upper lobe, middle lobe, and lower lobe superior segment at initial presentation (A), increased extent of consolidations with right-sided pleural effusion on the 10th day of hospitalization (B), and complete resolution after a 10-day treatment course (C). ELISA for (D) antigen was positive, leading to a diagnosis. After a 10-day treatment course with (E), the patient recovered completely without any sequelae.

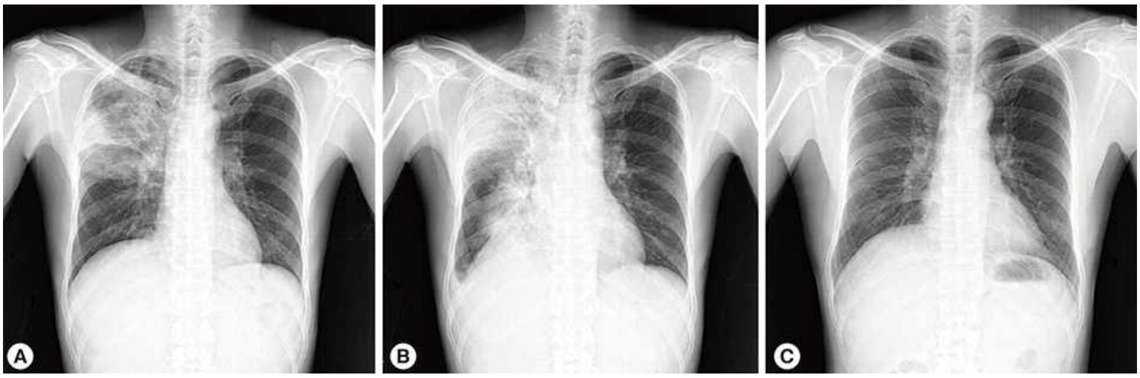

41. What is the parasite in (D)?

1) Anisakis simplex
2) Ascaris lumbricoides
3) Pneumocystis carinii
4) Echinococcus granulosus
5) Toxocara canis

A> 선충, 개회충, 판단형, 5

42. What is the drugs in (E)?

1) Chloroquine with primaquine
2) Albendazole with steroid
3) Praziquantel with amphotericin B
4) Metronidazole with amphotericin B
5) Trimethoxazole with amphotericin B

A> 선충, 개회충, 문제해결형 2

43. What may be a cause of infection to this case?

1) He has reared cows for 20 years.
2) He ate the raw beef frequently.
3) He enjoyed the vegetables every day.
4) He enjoyed eating raw sea fishes.
5) He reared dog at home.

A> 선충, 개회충, 암기형 2-->5

44. What is the blood smear finding of this patient?

1) Leukopenia
2) Eosinophilia
3) Anemia
4) Thrombocytopenia
5) Leukocytosis

A> 선충, 개회충, 암기형 2

45. What is the seropositive prevalence of this parasitic infection among Korean adults?

1) 1%
2) 2%
3) 3%
4) 4%
5) 5%

A> 선충, 개회충, 암기형 5

Case> A 59-year-old man presented at a hospital with fever and abdominal pain, which had persisted for 10 days. The patient alleged that he had managed his hypertension well, although complications of arterial hypertension such as ischemic heart disease or renal damage might have been present. On admission, the patient had impaired consciousness, shock, hypoxia and haziness in both lungs, jaundice, thrombocytopenia and disseminated intravascular coagulation, metabolic acidosis, and acute kidney injury. A peripheral blood smear (Fig.) and a rapid diagnostic test verified (A) infection. Ten hours after admission, hypoxia became more severe, despite providing maximal ventilatory support. The administration of (B), Extracorporeal membrane oxygenation, and continuous venovenous hemofiltration resulted in an improvement of his vital signs and laboratory findings. He was

discharged from the hospital 7 weeks later, without any sequelae.

Figure. Blood smear finding of patient who complains of fever and abdominal pain.

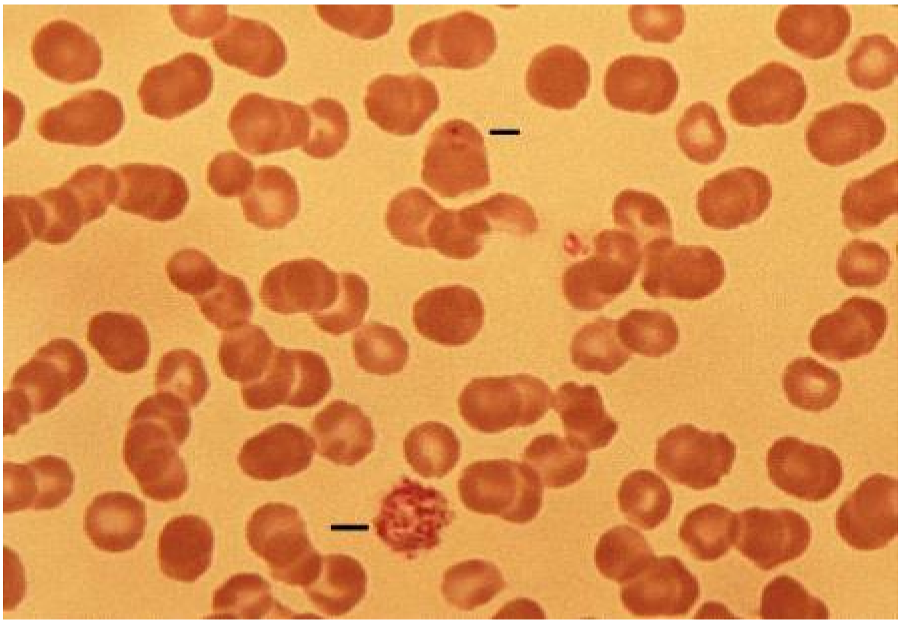

46. What is the (A)?

1) Plasmodium malariae
2) Plasmodium falciparum
3) Plasmodium ovale
4) Plasmodium vivax
5) Babesia microti

A> 원충, 말라리아, 판단형 4

47. What is the drugs in (B)

1) Chloroquine with primaquine
2) Albendazole with steroid
3) Praziquantel with amphotericin B
4) Metronidazole with amphotericin B
5) Trimethoxazole with amphotericin B

A> 원충, 말라리아, 문제해결형 1

48. What is the risk factor of multi-organ failure in this patient?

1) age

2) hypertension
3) ischemic heart disease
4) renal failure
5) sex

A> 원충, 말라리아, 암기형 1)

49. What is the name of vector of this pathogen in Korea?

1) Aedes togoi
2) Anopheles sinensis
3) Culex tritaeniorhyncus
4) Aedes aegypti
5) Anopheles gambiae

A> 원충, 말라리아, 암기형 2

50. In Figure what is the developmental stage of pathogen indicated by long bar?

1) ring form
2) trophozoite
3) merozoite
4) gametocyte
5) schizont

A> 원충, 말라리아, 판단형 5

Case> A 47-year-old man was hospitalized because of sharp epigastric pain and repeated vomiting after eating (A) 3 days previously. On admission, esophagogastroduodenoscopic examination revealed as Figure.

Figure. Esophagogastroduodenoscopic finding of a 47-year-old man.

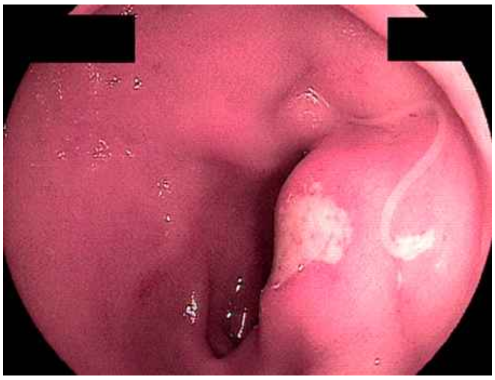

51. What is meal in (A)

1) Raw beef
2) Raw liver of cows
3) Vegetable
4) Raw enchovy
5) Raw fresh water fish

A> 선충, 고래회충, 판단형 4

52. What is the best treatment method for this patient?

1) Albendazole administration
2) Chloroquine administration
3) Praziquantel administration
4) Cauterization of worm
5) Removal of worm with endoscope

A> 선충, 고래회충, 문제해결형 5

53. What is the pathogen?

1) larva of *Anisakis simplex*
2) larva of *Ascaris lumbricoides*
3) larva of *Enterobius vermicularis*
4) larva of *Trichuris trichiura*
5) larva of *Toxocara canis*

A> 선충, 고래회충, 판단형. 1

Case> Following is the prevalence map of an intestinal parasite surveyed in 2012.

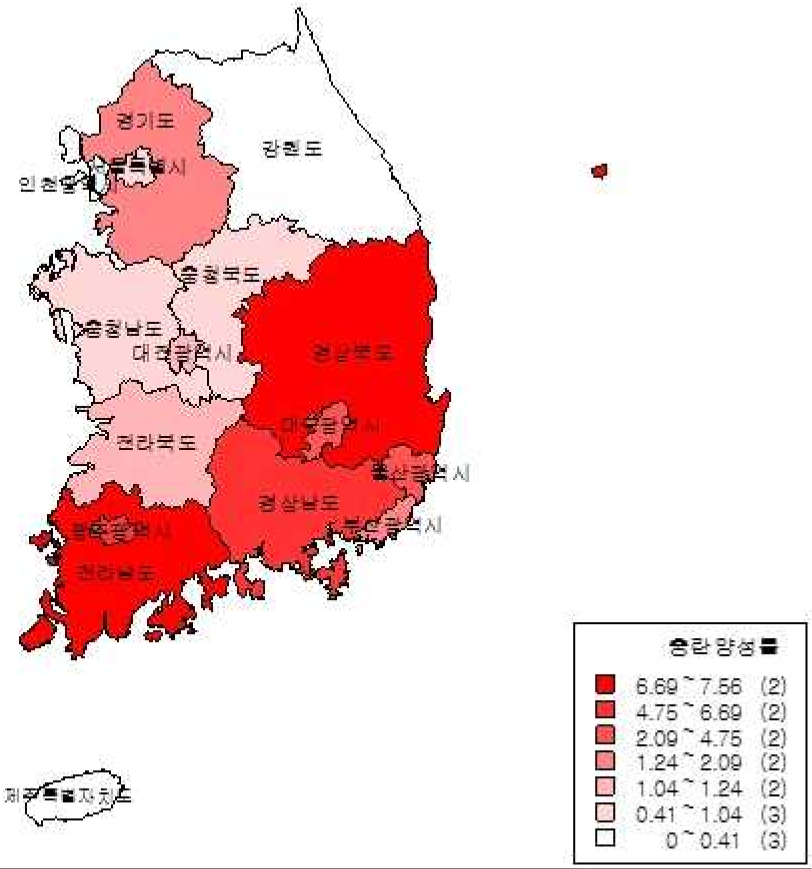

54. What is the name of parasite?

1) Enterobius vermicularis
2) Trichuris trichiura
3) Metagonimus yokogawai
4) Clonorchis sinensis
5) Ascaris lumbricoides

A> 흡충, 간흡충, 판단형 4

55. What is the mode of transmission of this parasite?

1) Soil transmitteed
2) Food-borne
3) Snail transmitted
4) Contact-borne
5) Vector-borne

A> 흡충, 간흡충, 암기형 3

56. Why is this parasitic infection still persistent in Korea?

1) There was a increased degree of pollution of river
2) There was no change of eating habit.
3) There was an increase of vector density.
4) The annual average temperature increase year by year.
5) There was no Government budget to eradicate this parasite.

A> 흡충, 간흡충, 암기형 2

57. What is the best drug for eradicating this parasitic infection?

1) Chloroquine
2) Albendazole
3) Praziquantel
4) Metronidazole
5) Trimethoxazole

A> 흡충, 간흡충, 문제해결형 3

Case 6> A previously healthy 25-year-old Haitian female, suffering only from headaches and back pain since delivery after an uneventful pregnancy 1 month previously, was admitted for sudden visual loss and impaired consciousness. She had no motor or sensory disturbance, but had complete visual loss with fixed, dilated pupils. Her body temperature was 38℃. MRI of the head was shown in Fig. Test (A) was done for diagnosis.

58. What is the MRI finding?

1) hydrocephalus and hyperintense calcified cyst
2) dilated lateral ventricles with a calcified cyst in the right occipital lobe
3) mass lesion compression of the cauda equina
4) Multiple hyperintense space occupying lesion
5) Cerebral edema

A> 조충, 유구낭미충, 판단형 1-->2

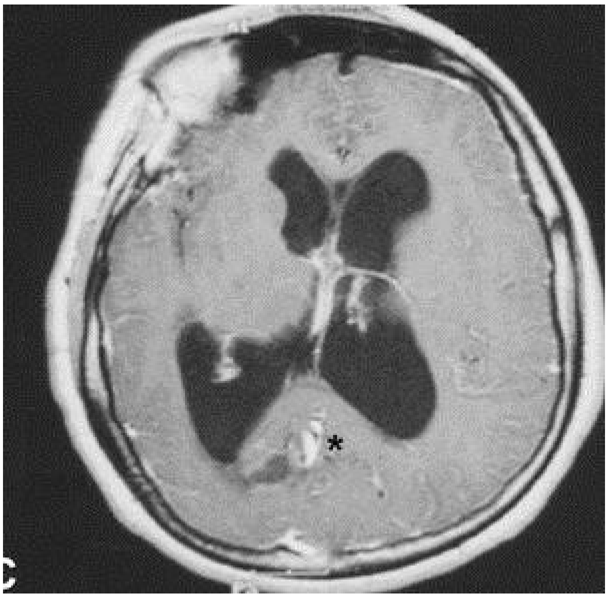

59. What is the most probable causative pathogen?

1) Ascaris lumbricoides
2. Taenia solium metacestode
3. Echinococcus multilocularis
4. Toxocara canis
5. Spirometra erinacei metacestode

A> 조충, 유구낭미충, 암기형 2

60. After the surgery, what is recommended as adjunctive chemotherapy?

1) Chloroquine
2) Primaquine
3) Praziquantel
4) Metronidazole
5) Trimethoxazole

A> 조충, 유구낭미충, 문제해결형 3

61. What is the mode of transmission to this patient?

1) She ate ham.
2) She ate raw beef
3) She lives besides an infected person with *Taenia solium*

4) She ate fresh water fish
5) She ate sea fish

A> 조충, 유구낭미충, 암기형 3

62. What is the test (B)?

1) ELISA
2) Indirect hemagglutination test
3) PCR
4) RT-PCR
5) Radioimmuno assay

A> 조충, 유구낭미충, 암기형 1

Case> A 48-year-old man identified a skin rash that occurred three weeks ago and spread throughout his body. The biopsy result is show in the Figure.

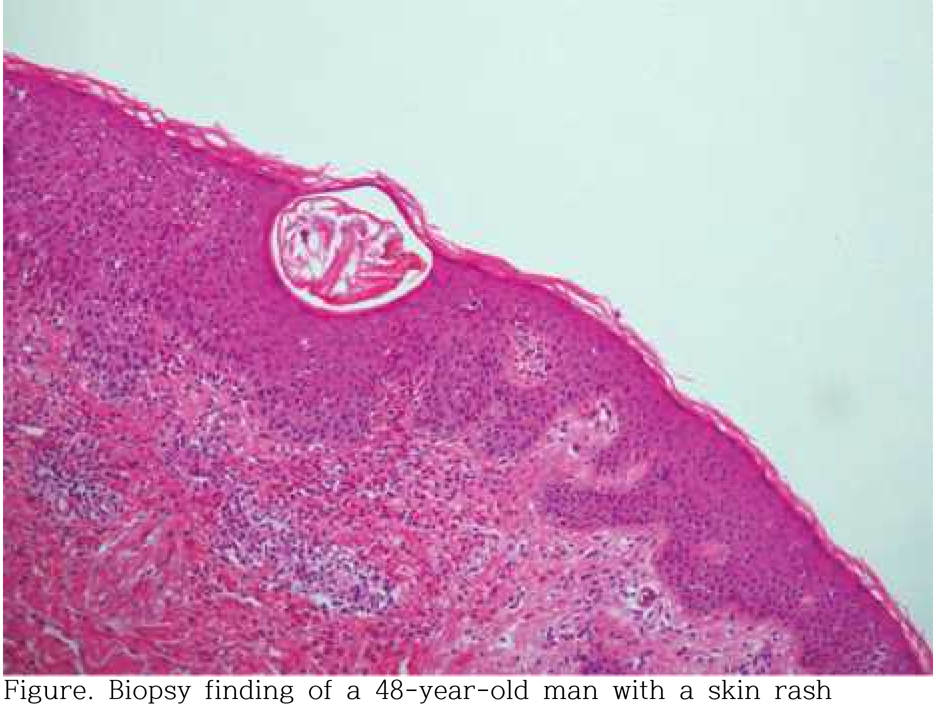

Figure. Biopsy finding of a 48-year-old man with a skin rash

63. What is the diagnosis?

1. Scabies
2. Toxocariasis

3. Paragonimiasis
4. Sparganosis
5. Cysticercosis

A> 절지동물,옴, 판단형 1

64. What is the therapeutic drug?

1. Crotamiton
2. Praziquantel
3. Albendazole
4. Chloroquine
5. Metronidazole

A> 절지동물,옴. 문제해결형 1

Case> A woman who lived in the Republic of Upper Volta, Africa returned home and delivered baby. Baby has been hospitalized with high fever, nervousness, pale, jaundice, and hepatosplenomegaly since the 39th day after birth. Her laboratory findings showed the hypoglycemia, bilirubinemai, increased SGOT/SGPT. Peripheral blood findings are shown in the Figure.

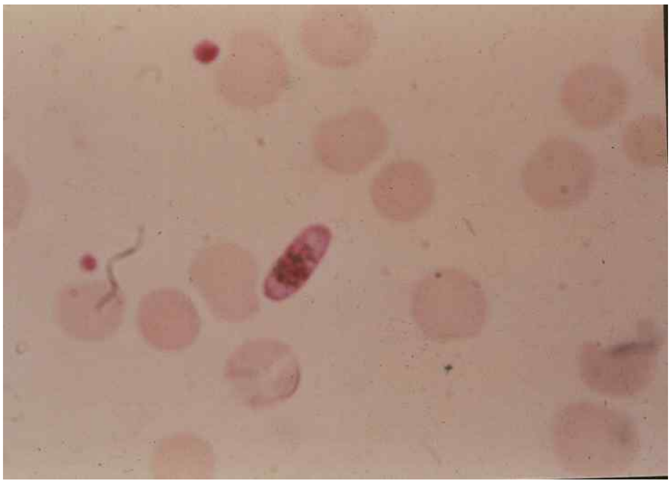

Figure. Peripheral blood findings of the 39 day-old baby.

65. What is the causative pathogen?

1) Plasmodium faciparum
2) Plasmodium vivax
3) Plasmodium ovale
4) Plasmodium malariae
5) Babesia spp.

정답> 원충, 말라리아. 판단형 1

66. The first treatment drug for the baby was administered, but the high fever continued. What is the most appropriate drug afterwards?

1) Mefloquine
2) Primaquine
3) Praziquantel
4) Albendazole
5) Metronidazole

정답: 원충, 말라리아. 문제해결형 1

67. What is the disease of which vector is *Anopheles sinensis*?

1) Plasmodium vivax malaria
2) Brugia malayi infection
3) Scrub typhus
4) Japanese B encephalitis
5) Lyme disease

답> 절지동물, 모기, 암기형 1

case> A 17-year-old boy visited the dermatologist due to itching in his fingers. Lesion is shown in Figure 1. Worm was found as in Figure 2 after biopsy.

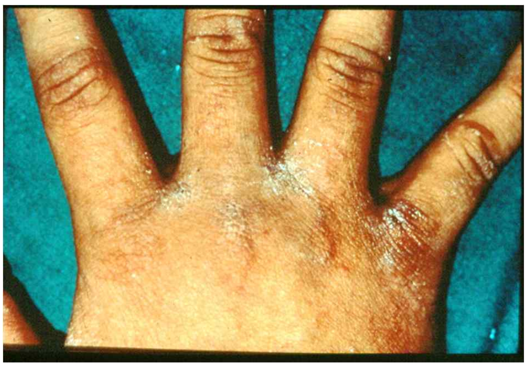

Figure 1. Skin lesion

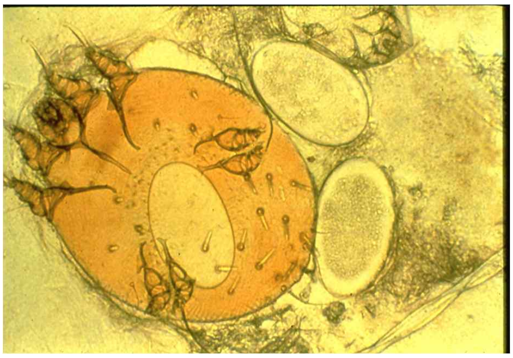

Figure 2. Worm

68. What is the causative worm?

1) Sarcoptes scabiei
2) Pthirus pubis
3) Pediculus humanus corporis
4) Dermatophagoides farinae
5) Leptotrombidium pallidum

답> 절지동물, 옴, 판단형 1

69. What is the therapeutic drug?

1) Praziquantel
2) Albendzole
3) Crotamiton
4) Metronidazole
5) Chloroquine

답> 3

70. What is the most common allergen in Korea?

1) Mold
2) Pollen
3) House dust mites

4) bed bug
5) egg

답> 절지동물, 진드기, 암기형 3

71. What is the vector of Tsutsugamushi disease?

1) Sarcopetes scabiei
2) Leptotrombidium pallidum
3) Aedes togoi
4) Haemaphysalis longicornis
5) Culex tritaeniorhyncus

답> 절지동물, 진드기, 암기형 2

72. What is the endemic disease in Korea of which pathogen was transmitted through transovarian transmission in the vector?

1) Malaria
2) Malayian filariasis
3) Tsutsugamushi disease
4) Leptospirosis
5) Lyme disease

답> 절지동물, 진드기, 암기형 3

case> The following worm was found in a pubic hair of 26-year-old female.

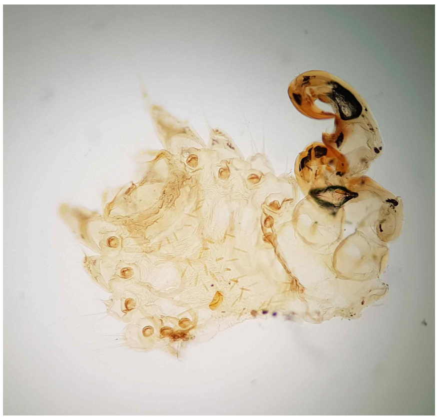

73. What is the name of the worm?

1) Sarcoptes scabiei
2) Pthirus pubis
3) Pediculus humanus corporis
4) Dermatophagoides farinae
5) Leptotrombidium pallidum

답> 절지동물,사면발이, 판단형, 2

74. What is the mode of transmission of this worm?

1) Fish-borne
2) Water-borne
3) Sexual contact
4) Air-borne
5) Food-borne

답> 절지동물,사면발이, 암기형 3

case> From hair of a five-year-old kindergarten child, the following worms were found.

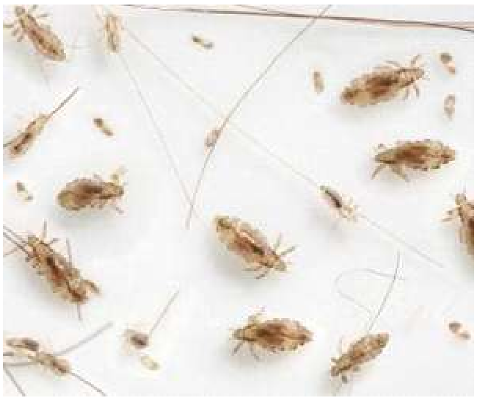

75. What is the name of worm?

1) Sarcoptes scabiei
2) Pthirus pubis
3) Pediculus humanus capitis
4) Dermatophagoides farinae
5) Leptotrombidium pallidum

답>절지동물, 머릿니 판단형 3

76. What is the treatment of choice?

1) Hair shampooing every day
2) Praziquantel administration.
3) Changing underwear every day.
4) Brushing hair every day
5) Apply Crotamiton ointment in the hair

답>절지동물, 머릿니 문제해결형 1

77. What is the vector of Severe Fever with Thrombocytopenia Syndrome in Korea?

1) H*aemaphysalis longicornis*
2) *Pediculus humanus capitis*
3) *Anopheles sinensis*
4) *Culex tritaeniorhynchus*
5) *Aedes togoi*

답> 절지동물, 진드기, 암기형 1

78. What is the vector of Lyme disease in Korea?

1) *Haemaphysalis japonica*
2) *Pediculus humanus*
3) *Ixodes nipponensis*
4) *Aedes togoi*
5) *Anopheles sinensis*

답> 절지동물, 참진드기, 암기형 3

79. What is the vector of anaplasmosis in Korea?

1) *Aedes togoi*
2) *Ixodes nipponensis*

3) *Culex tritaeniorhyncus*
4) *Anopheles sinensis*)
5) *Leptotrombidium pallidum*

답> 절지동물, 참진드기 암기형 2

List of license for figures and tables used for parasitology examination. Use of following tables and figures are not for commercial purpose but educational purpose.

- Case for No. 1. Case is virtual and the figure is from slide for educational purpose.
- Case for No. 10. Tables are reproduced from Korean J Parasitol. 2014;52 (5): 551-555.https://doi.org/10.3347/kjp.2014.52.5.551 according to the CC-BY-NC license.
- Case for No. 13: Figures are reproduced from Ann Dermatol. 2013 Feb;25(1):80-3. https://doi.org/10.5021/ad.2013.25.1.80 according to the CC-BY-NC license.
- Cass for No. 16. Figure is from sectional slide of trichinellosis case not published, photographed by the author.
- Case for No. 18. Figure is reproduced from Dermanet. 2003. https://dermnetnz.org/topics/cutaneous-larva-migrans according to the CC-BY-NC-ND license.
- Case for No. 19. Figure is reproduced from Yonsei Med J. 1998. Available from: http://doi.org/10.3349/ymj.1998.39.2.184 according to the CC-BY-NC license.
- Case for No. 20. Figure is from slides for education.
- Case for No. 21. Two figures are from case unpublished.
- Case for No. 23. Case is reproduced from Yonsei Med J. 1998;39(5):478-484. http://doi.org/10.3349/ymj.1998.39.5.478 according to the CC-BY-NC license, but figure is from slide for educational purpose.
- Case for No. 24. Figures are reproduced from Korean J Parasitol. 2003;41(2):97-100. http://doi.org/10.3347/kjp.2003.41.2.97 according to the CC-BY-NC license.
- Case for No. 32. Figure is reproduced from: Korea Centers for Disease Control and Prevention. Korea Association of Health Promotion: Prevalence of intestinal parasites in Korea - The 8th Report. Seoul, Korea. 2012 according to the CC-BY license of the Korean Government.
- Case for No. 33. Figure was photographed by the author.
- Case for No. 34. Figures are reproduced from: Korean J Parasitol. 2009 Sep;47(3):275-80. http://doi.org/10.3347/kjp.2009.47.3.275 according to the

CC-BY-NC license the CC-BY-NC license.

- Case for No. 37. Figure was photographed by the author.
- Case for No. 41. Figures were reproduced from: Korean J Parasitol. 2014;52(4):425-428. http://doi.org/10.3347/kjp.2014.52.4.425 according to the CC-BY-NC license the CC-BY-NC license.
- Case for No. 46. Case is reproduced from Korean J Parasitol. 2013 Oct;51(5):551-5. http://doi.org/10.3347/kjp.2013.51.5.551 according to the CC-BY-NC license the CC-BY-NC license. Figure was not included in the article. Another figure is used taken by the author
- Case for No. 51. Figure is reproduced from Chonnam Med J. 2012;48(1):73-75. http://doi.org/10.4068/cmj.2012.48.1.73 according to the CC-BY-NC license
- Case for No. 54. Figure is reproduced from Korea Centers for Disease Control and Prevention. Korea Association of Health Promotion: Prevalence of intestinal parasites in Korea - The 8th Report. Seoul, Korea. 2012 according to the CC-BY license of the Korean Government.
- Case for No. 58. Figure is reproduced from J Clin Neurol. 2014;10(4):363-366. http://doi.org/10.3988/jcn.2014.10.4.363 according to the CC-BY-NC license
- Case for No. 63. Figure is reproduced from Lab Med Online. 2012;2(4):240-241. https://doi.org/10.3343/lmo.2012.2.4.240 according to the CC-BY-NC license
- Case for N0. 65. Case is reproduced from Kisaengchunghak Chapchi. 1984;22(1):72-77. http://doi.org/10.3347/kjp.1984.22.1.72 according to the CC-BY-NC license the CC-BY-NC license. Figure is from slide photographed by the author.
- Case for No. 68. The case is an unpublished one. Figures are donated from a dermatologist in Korea for educational purpose.
- Case for No. 73. Figure was photographed by the author.
- Case for No. 75. Figure was photographed by the author.
