# Supplementary material for: Are ChatGPT’s knowledge and interpretation ability comparable to those of medical students in Korea for taking a parasitology examination?: a descriptive study
Source: J Educ Eval Health Prof. 2023 Jan 11;20:1. doi: 10.3352/jeehp.2023.20.1 (PMC9905868; doi:10.3352/jeehp.2023.20.1)
Supplement: Supplementary file 3 — Supplement 2. ChatGPT’s responses to 79 items from a parasitology examination taken by first-year medical students at Hallym University on December 12, 2022, inputted on January 1, 2023, by the author. Figures and tables are removed and explained in the item stem. Explanations of the options selected by ChatGPT are also included. [file jeehp-20-1-suppl2.pdf]

Supplement 2. Seventy-nine parasitology examination items, which medical students took on December 12, 2022, at Hallym University through computer-based testing, input to ChatGPT and its answers on January 1, 2023. The figures and tables were redescribed and input.

Case> A 45-year-old female patient visited a nearby hospital for abdominal discomfort that occurred two weeks before her visit to the hospital. She was diagnosed with urinary tract infection, and was treated with antibiotics. After treatment, she complained of minor gastrointestinal symptoms continuously. Gastric endoscopy was performed but no special findings were observed in the upper gastrointestinal endoscopy. The colonoscopy was done also. Result of colon mucous membrane surface biopsy were shown in the figure. Parasitic pathogen can be seen in the black circle marking.

1. What is the scientific name of this parasite? Choose one best answer out of the below 5 options:

1) Entamoeba histolytica
2) Entamoeba coli
3) Giardia lamblia
4) Endolixmax nana
5) Trichomonas vaginalis

It is not possible for me to accurately identify the parasite based on the information provided. The scientific name of a parasite can often be determined through laboratory testing and examination under a microscope by a trained medical professional. It is important for the patient to follow the recommended treatment plan provided by their healthcare provider in order to address any potential infections or other health concerns.

2. What is the therapeutic drug for amoebiasis out of below 5 opptions?

1) Albendazole
2) Praziquantel
3) Primaquine
4) Metronidazole
5) Ivermectin

Amoebiasis is an infection caused by the parasite Entamoeba histolytica. The most commonly used

therapeutic drug for amoebiasis is metronidazole. This drug is effective at killing the parasite and eliminating the infection. Other options that may be used to treat amoebiasis include paromomycin, iodoquinol, and diloxanide furoate. Of the options listed in your question, metronidazole (option 4) is the most effective treatment for amoebiasis.

3. What is the patient's past history for cause of amoebiasis out of below 5 options? Choose one best option.

1) Eating raw pork

2) Eating raw fresh water fish

3) Eating raw beef

4) Eating vegetable during the travel to Vietnam

5) Eating raw raw oyster

It is not possible to determine the patient's past history or the cause of their amoebiasis based on the information provided. Amoebiasis can be caused by consuming contaminated food or water, or by coming into contact with infected feces. All of the options listed in your question (eating raw pork, raw fresh water fish, raw beef, eating vegetables during travel to Vietnam, or eating raw oysters) could potentially lead to amoebiasis if the food or water is contaminated with the parasite. However, without more information about the patient's medical history and specific risk factors, it is not possible to accurately determine the cause of their infection.

4. What is the mechanism of causing lesion in the intestinal amoebiasis out of below 5 option? Choose one best option.

1) Intrusion to crypt

2) Covering villous membrane

3) Invasion to mucosa

4) Allergic reaction to the excretory protein of the parasite

5) Increased number of the secretory gland

In intestinal amoebiasis, the parasite Entamoeba histolytica causes lesions by invading and damaging the mucosa (option 3). The parasite attaches to the intestinal mucosa and produces enzymes that digest the tissue, leading to inflammation, ulceration, and tissue destruction. The mechanism of lesion formation in amoebiasis does not involve intrusion into the crypts (option 1),

covering of the villous membrane (option 2), allergic reactions to the excretory protein of the parasite (option 4), or an increase in the number of secretory glands (option 5).

Case> A 45-year-old man who complained of the abdominal pain and diarrhea visited an outpatient clinic. The diameter of the parasite was 5 micrometers by the Modified acid fast stain of stool. Oocyst was stained as red with 4 nuclei. What is the most probable name of causative parasite out of below 5 option?

1) Microsporidia spp.
2) Cyclospora spp.
3) Cryptosporidium spp.
4) Isospora spp.
5) Giardia spp.

Based on the information provided, the most probable name of the causative parasite in this case is Cryptosporidium spp. (option 3). Cryptosporidium is a parasite that can cause abdominal pain and diarrhea in humans. It is characterized by small, oocyst-shaped spores that are 5 micrometers in diameter and have 4 nuclei, which are stained red by the Modified acid fast stain. The other options listed (Microsporidia spp., Cyclospora spp., Isospora spp., and Giardia spp.) are also parasites that can cause abdominal pain and diarrhea, but they do not match the description provided in the case.

6. What is the best stain method of stool used in for diagnosis of cryptosporidiosis out of below 5 options?

1) Modified acid-fast stain
2) Giemsa stain
3) Iron-hematoxylin stain
4) Hmatoxylin-eosin stain
5) Trichrome stain

The best stain method for the diagnosis of cryptosporidiosis is the modified acid-fast stain (option 1). This stain is specifically designed to detect the presence of Cryptosporidium parasites in stool samples. It involves the use of an acid-fast dye, such as auramine O or fluorescein, which is resistant to decolorization by acid or alcohol. The modified acid-fast stain is highly sensitive and specific for the detection of Cryptosporidium and is the recommended method for the diagnosis

of cryptosporidiosis. The other options listed (Giemsa stain, iron-hematoxylin stain, hematoxylin-eosin stain, and trichrome stain) may be useful for the diagnosis of other types of infections, but they are not as effective at detecting Cryptosporidium.

7. What is the most appropriate treatment method of cryptosporidosis out of belwo 5 options?
1) Prescription of praziquantel
2) Prescription of albendazole
3) Prescription of chloroquine
4) Prescription of metronidazole
5) Conservative measure

The most appropriate treatment for cryptosporidiosis is prescription of nitazoxanide (option 5). Nitazoxanide is an antiparasitic medication that is specifically indicated for the treatment of infections caused by Cryptosporidium. It works by inhibiting the growth and reproduction of the parasite, and has been shown to be effective at reducing the severity and duration of cryptosporidiosis. The other options listed (praziquantel, albendazole, chloroquine, and metronidazole) are not effective at treating cryptosporidiosis and are not recommended as treatment options. In some cases, supportive care, such as hydration and electrolyte replacement, may be necessary to manage the symptoms of the infection. However, the primary treatment for cryptosporidiosis is the use of an antiparasitic medication such as nitazoxanide.
[Author's comment: Although nitazoxanide is mentioned as a therapeutic drug, it is not widely used in the clinical field. Usually, immunocompetent persons do not need to be prescribed because it is a self-recovering disease for them. As for immunocompromised patients, nitazoxanide was not found to be effective. Therefore, the treatment of choice is the conservative treatment.]

8. What is the highly specific diaganostic methods of cryptosporidiosis besides of stool exam out of below 5 options?
1) Indirect immuno-fluorescent assay
2) Polymerase chain reaction
3) Enzyme linked immunosorbent assay
4) Western blot
5) Northern blot

The highly specific diagnostic methods for cryptosporidiosis besides stool examination include polymerase chain reaction (PCR) (option 2) and indirect immunofluorescent assay (option 1).

PCR is a molecular technique that involves amplifying small amounts of DNA from the Cryptosporidium parasite. It is highly sensitive and specific for the detection of Cryptosporidium and can be used to diagnose infection in a variety of clinical specimens, including stool, duodenal aspirates, and respiratory secretions.

Indirect immunofluorescent assay (IFA) is a laboratory technique that involves using fluorescently labeled antibodies to detect the presence of Cryptosporidium antigens in a sample. It is highly sensitive and specific for the detection of Cryptosporidium and can be used to diagnose infection in a variety of clinical specimens, including stool, duodenal aspirates, and respiratory secretions.

The other options listed (enzyme-linked immunosorbent assay (ELISA), western blot, and northern blot) may be useful for the diagnosis of other types of infections, but they are not as effective at detecting Cryptosporidium.
[Author's comment: PCR is the specific diagnostic methods beside stool exam with modified acid-fast stain]
9. What is the most frequent source of infection in cryptosporidiosis patient? Choose one best answer out of below 5 options.
1) Eating raw pork
2) Eating raw fresh water fish
3) Eating raw beef
4) Eating vegetable
5) Eating raw oyster

The most frequent source of infection in cryptosporidiosis is consumption of contaminated water (option 4). Cryptosporidium is a waterborne parasite that is transmitted through the ingestion of fecally contaminated water or food. It is commonly found in surface water sources, such as lakes, streams, and rivers, and can also be transmitted through contact with infected animals, such as cattle and sheep. The other options listed (eating raw pork, raw fresh water fish, raw beef, or raw oysters) could potentially lead to cryptosporidiosis if the food or water is contaminated with the parasite,

but the most common source of infection is contaminated water.

[Autor's comment: ChatGTP can not select the best answer out of presented options. It needs more training]

10. What are the most common symptoms of men's trichominasis out of below 5 options?

1) Penile discharge
2) Penile tingling
3) Pain during urination
4) Lower abdominal pain
5) Frequent urination

The most common symptoms of trichomoniasis in men are penile discharge (option 1) and pain during urination (option 3). Trichomoniasis is a sexually transmitted infection caused by the parasite Trichomonas vaginalis. In men, the infection is typically asymptomatic, but some men may experience symptoms such as penile discharge, pain during urination, and a burning sensation in the genital area. The other options listed (penile tingling, lower abdominal pain, and frequent urination) are not typically associated with trichomoniasis in men.

[Autor's comment: ChatGTP can not select the best answer out of the presented options. Also, it can not interpret the data in the item stem. It needs more training]

11. What is the therapeutic drug for above Trichomonas positive visitors? Choose one best option out of below 5 options.

1) Albendazole
2) Praziquantel
3) Primaquine
4) Metronidazole
5) Ivermectin

The most appropriate therapeutic drug for the treatment of trichomoniasis is metronidazole (option 4). Metronidazole is a powerful antimicrobial agent that is effective at killing the Trichomonas vaginalis parasite and eliminating the infection. It is the recommended first-line treatment for trichomoniasis and is usually administered as a single oral dose. The other options listed (albendazole, praziquantel, primaquine, and ivermectin) are not effective at treating

trichomoniasis and are not recommended as treatment options. It is important to note that both sexual partners should be treated to ensure that the infection is completely eliminated.

12. What is the mechanism causing lesion in the vagina of Trichomonas vaginalis infected female? Choose one best option out of below 5 options.

1) Harboring in the vaginal epithelium
2) Covering vaginal epithelium so that increasing secretion
3) Changing the vaginal pH into acidic status
4) Inhibiting the proliferation of the vaginal epithelium
5) Inducing chemolucence of neutrophil by the excretory-secretory protein

The mechanism causing lesions in the vagina of a female infected with Trichomonas vaginalis is infiltration and damage to the vaginal epithelium (option 1). Trichomonas vaginalis is a parasite that can infect the genital tract and cause inflammation and tissue damage. The parasite attaches to the vaginal epithelium and produces enzymes that digest the tissue, leading to inflammation, ulceration, and tissue destruction. The mechanism of lesion formation in trichomoniasis does not involve covering of the vaginal epithelium (option 2), changing the vaginal pH (option 3), inhibiting the proliferation of the vaginal epithelium (option 4), or inducing chemoluminescence of neutrophils (option 5).

[Author's comment: The most critical mechanism of pathogensis is the inducing chemolucence of a neutrophil by the excretory-secretory protein]

13. What is the causative agent of cutaneous leishmaniasis? Choose one best option out of below 5 options.

1) Leishmania braziliensis
2) Leishmania donovani
3) Trypanosoma brucei gambiense
4) Trypanosoma brucei rhodesiense
5) Trypanosoma cruzi

The causative agent of cutaneous leishmaniasis is Leishmania braziliensis.

14. What is the drug for cutaneous leishmaniasis? Choose one best option out of below 5 options.

1) sodium stibogluconate injection to the lesion

2) Praziquantel

3) Primaquine

4) Metronidazole

5) Ivermectin

The most common treatment for cutaneous leishmaniasis is sodium stibogluconate injection to the lesion. Other treatments that may be used include:

Amphotericin B

Miltefosine

Paromomycin

It is important to note that the treatment for cutaneous leishmaniasis can vary depending on the specific type of Leishmania that is causing the infection, the severity of the infection, and the overall health of the patient. It is important to follow the recommended treatment plan provided by a healthcare professional in order to effectively treat the infection.

15. What is the vector of cutaneous leishmaniasis out of below 5 options?

1) sand fly

2) tsetse fly

3) kissing bug

4) mite

5) mosquito

The vector of cutaneous leishmaniasis is the sand fly. Sand flies are small, biting insects that can transmit the Leishmania parasites that cause cutaneous leishmaniasis. They are found in many parts of the world, including Central and South America, Africa, the Middle East, and parts of Asia. When a sand fly bites an infected person or animal, it can pick up the parasites and then transmit them to another person or animal when it bites again. It is important to take steps to protect yourself from sand fly bites in areas where cutaneous leishmaniasis is common, such as using insect repellent and wearing long sleeves and pants.

Case> In March 2003, 13 adults went hunting in Inje, Gangwon Province, and ate wild boars and badgers captured. They complained of high fever, muscle pain, and facial edema. Tissue biopsy

findings showed the coiled larvae in muscle.
16. What is the causative agent?
1) Trichinella spiralis
2) Gnathostoma spinigerum
3) Anisakis simplex larvae
4) Toxocara canis larvae
5) Brugia malayi

The causative agent in this case is Trichinella spiralis. Trichinella spiralis is a parasitic roundworm that can cause a disease called trichinosis. The infection is usually transmitted to humans through the consumption of undercooked or raw pork products that contain the larvae of the worm. Symptoms of trichinosis can include fever, muscle pain, and facial edema, as well as other symptoms such as nausea, vomiting, and diarrhea. Treatment for trichinosis typically involves the use of medications to kill the worms and reduce inflammation, and can also include supportive care to manage symptoms. It is important to thoroughly cook pork products in order to prevent infection with Trichinella spiralis.
17.What is the therapeutic drug to patients with trichinellosis out of below 5 options?
1) Levamisole
2) Ivermectin
3) Praziquantel
4) Diethyl carbamazine
5) Albendazole

The most common therapeutic drug for trichinellosis is albendazole. Albendazole is an antiparasitic medication that is effective at killing the worms that cause trichinellosis. It is typically taken by mouth for a period of several weeks in order to completely eradicate the infection. Other medications that may be used to treat trichinellosis include mebendazole, levamisole, and ivermectin. It is important to follow the treatment plan provided by a healthcare professional in order to effectively treat the infection and prevent complications.

Walking barefoot along the coast resulted in the serpiginous rash on the the back of his foot .
18. What is the most probable causative agent out of below 5 options?

1) Ancylostoma duodenale

2) Ascaris lumbricoides

3) Trichuris trichiura

4) Oncocerca volvulus

5) Brugia malayi

The most probable causative agent in this case is Ancylostoma duodenale. Ancylostoma duodenale, also known as the "old world hookworm," is a type of parasitic worm that can infect humans and cause a disease called ancylostomiasis. One of the common symptoms of ancylostomiasis is a serpiginous rash, which is a rash that is characterized by a winding or serpentine pattern. The infection is typically transmitted through contact with soil or sand that is contaminated with the worms, and can be prevented by wearing shoes or other protective footwear when walking in areas where the worms are common. Treatment for ancylostomiasis typically involves the use of antiparasitic medications to kill the worms and prevent further damage to the body.

A 38-year-old man visited an outpatient clinic in Gabon, Africa, with the diffuse welling of the right hand arm.

19. What is the causative agent?

1) Wucheria bancrofti

2) Bruia malayi

3) Onchocerca volvulus

4) Loa loa

5) Dirofilaria immitis

The causative agent in this case is likely to be Wuchereria bancrofti. Wuchereria bancrofti is a parasitic worm that can cause a disease called filariasis. One of the common symptoms of filariasis is the development of a diffuse swelling in the arms and legs, known as lymphatic elephantiasis. The infection is transmitted to humans through the bites of infected mosquitoes, and is most common in tropical and subtropical areas of Africa, Asia, and the Americas. Treatment for filariasis typically involves the use of antiparasitic medications to kill the worms and prevent further damage to the body. It is important to take steps to prevent mosquito bites in areas where filariasis is common, such as using insect repellent and wearing protective clothing.

[Author" comment: The case is from https://pc.eymj.org/Synapse/Data/PDFData/0069YMJ/ymj-39-184.pdf. If examinees do not know this information, it will be challenging to anwer this item]

20. What is the name of parasite and its developmental stage, that migrates in the conjunctiva of the right eye of man? Choose one best option out of below 5 options.
1) Trichinella spiralis larva
2) Toxocara canis larva
3) Thelazia callipaeda adult
4) Brugia malayi filaria
5) Anisakis simplex larva

The name of the parasite that migrates in the conjunctiva of the eye is Thelazia callipaeda, and the developmental stage is the adult. Thelazia callipaeda is a type of parasitic worm that can infect humans and cause a disease called thelaziasis. The worms typically infect the eyes, and can cause symptoms such as irritation, redness, and discharge. The infection is transmitted to humans through the bites of infected flies, and is most commonly found in parts of Europe, Asia, and North Africa. Treatment for thelaziasis typically involves removing the worms from the eye and using medications to kill any remaining worms and reduce inflammation. It is important to protect the eyes from fly bites in areas where thelaziasis is common, such as by wearing protective eyewear or using insect repellent.
Case> A 72-day-old girl died after being hospitalized in an intensive care unit due to breathing difficulties. The result of observing Leukostat stain by smear printing the autopsy lung tissue showed the cyst of 7 micrometer in diameter with 8 sporozoites. The findings of the postmortem lung showed the consolidated lung.
21. What is the causative agent? Choose one best answer out of below 5 options.
1. Cryptosporidium parvum
2. Toxoplasm gonddii
3. Giardia lamblia
4. Pneumocystis carinii
5. Naegleria fowleri

Based on the description of the cyst, the most likely causative agent in this case is Cryptosporidium

parvum. This is a type of parasite that can cause gastrointestinal illness and can be transmitted through contaminated water or food. The presence of sporozoites, which are a stage in the life cycle of Cryptosporidium, further supports this diagnosis. However, it is important to note that additional testing, such as PCR or culture, would be necessary to confirm the diagnosis.

22. What is the drug of choice for pneumocystis carinii infection? Choose one best answer out of below 5 options.

1. Trimethoprim / sulfamethoxazole
2. Metronidazole
3. Albendazole
4. Praziquantel
5. Primaquine

The drug of choice for pneumocystis carinii infection is trimethoprim/sulfamethoxazole (also known as co-trimoxazole). This combination of drugs is highly effective against Pneumocystis jirovecii, the causative agent of pneumocystis pneumonia. It is usually given in a course of treatment lasting several weeks. Other drugs, such as pentamidine and atovaquone, may also be used as alternatives in some cases. It is important to note that treatment should be initiated as soon as possible in order to improve the chances of a successful outcome.

Case> A 7-year-old child had a torn left eyebrow, and after being sutured, a kidney-sized nodule developed in the wound and was treated with antibiotics, fungicides and steroids for 42 days. In the skin biopsy, the amoeboid trophozoite of 20 micrometer in diameter with multiple spine-like processes, was observed. While the wound was progressing, the child was hospitalized due to vomiting, lack of appetite, and a mild fever. The cerebrospinal fluid was clear, with negative gram-stain, bacterial culture, and Indian ink. On the 10th day of hospitalization, brain CT imaging showed the dense density in the left temporal lobe. The child became unconscious and died within a day later.

23. What is the causative agent of this fatal case? Choose one best answer out of below 5 options.

1. Cryptosporidium parvum
2. Toxoplasm gonddii
3. Giardia lamblia
4. Pneumocystis carinii

5. Acanthamoeba culbertsoni

Based on the description of the amoeboid trophozoite observed in the skin biopsy and the CT imaging findings, the most likely causative agent in this case is Acanthamoeba culbertsoni. This is a type of free-living amoeba that can cause a rare but potentially serious infection called granulomatous amoebic encephalitis (GAE). GAE typically occurs when the amoeba infects the central nervous system, often through a break in the skin or mucous membranes. The infection can cause symptoms such as vomiting, lack of appetite, and fever, and can progress rapidly to coma and death if left untreated. It is important to note that additional testing, such as PCR or culture, would be necessary to confirm the diagnosis.

Case> A 68-year-old woman residing in Busan, Korea, had epigastric pain with severe vomiting about 5 hours after eating meal. Four worms penetrating the gastric mucosae in the great curvature of the middle body and fundus were found and removed during gasteroendoscopic examination. Worm is 1.2 cm in length and 0.05 cm in width and white in color.

24. What is the causative pathogen? Choose one best answer out of below 5 options.

1) Larvae of Spirometra erinacei

2) Larvae of Taenia solium

3) Larvae of Toxocara canis

4) Larvae of Anisakis simplex

5) Larvae of Ascaris lumbricoides

Based on the description of the worms found in the patient's stomach, the most likely causative pathogen is Larvae of Anisakis simplex. This is a type of parasitic nematode that can cause a condition called anisakiasis, which is characterized by the presence of larvae in the gastrointestinal tract. The larvae can be transmitted to humans through ingestion of undercooked or contaminated fish or seafood, and can cause symptoms such as abdominal pain and vomiting. It is important to note that additional testing, such as PCR or microscopy, would be necessary to confirm the diagnosis.

[Author's comment: Incorrecte explanation was added. PCR is not necessary to diagnose the anisakiasis]

A 68-year-old woman residing in Busan, Korea, had epigastric pain with severe vomiting about 5 hours after eating meal. Four worms penetrating the gastric mucosae in the great curvature of the

middle body and fundus were found and removed during gasteroendoscopic examination. Worm is 1.2 cm in length and 0.05 cm in width and white in color. What is the most probable meal that the patient ingested in this case? Choose one best answer out of below 5 options.

1) raw beef
2) raw anchovy
3) raw snake
4) vegetable
5) raw cow liver

Based on the description of the worms found in the patient's stomach and the most common sources of Anisakis simplex, the most probable meal that the patient ingested in this case is raw anchovy. Anisakis simplex is a type of parasitic nematode that can infect various species of fish and seafood, and it is most commonly transmitted to humans through ingestion of undercooked or contaminated fish or seafood. Other common sources of Anisakis infection include squid, mackerel, and sardines. It is important to note that additional testing, such as PCR or microscopy, would be necessary to confirm the diagnosis.

[Author's comment: Incorrecte explanation was added. PCR is not necessary to diagnose the anisakiasis]

26. Which is the correct description of the scientific name of the parasite? Choose one best answer out of below 5 options.

1) Toxocara Canis
2) To. canis
3) TOXOCARA CANIS
4) T. Canis
5) T. c.

The correct description of the scientific name of the parasite Toxocara is Toxocara canis. In scientific nomenclature, the first letter of the genus name is capitalized and the entire species name is lowercase. In this case, "Toxocara" is the genus name and "canis" is the species name. The correct way to abbreviate the name would be "T. canis" or "T. c."

[Author's comment: Correct answer is 3, Large character and small character was not discriminated by ChatGTP]

27. What is the mode of transmission of Enterobius vermicluaris? Choose one best answer out of below 5 options.

1) snail-transmitted

2) soil-transmitted

3) arthropod-borne

4) contact-borne

5) food-borne

The mode of transmission of Enterobius vermicularis, also known as pinworm, is contact-borne. This means that it is transmitted through direct contact with an infected person, such as through shaking hands or sharing personal items. It is not transmitted through snails, soil, arthropods, or food.

28. What is the mode of transmission of Ascaris lumbricoides? Choose one best answer out of below 5 options.

1) snail-transmitted

2) soil-transmitted

3) arthropod-borne

4) contact-borne

5) food-borne

The mode of transmission of Ascaris lumbricoides, also known as roundworm, is soil-transmitted. This means that it is transmitted through contact with contaminated soil or food, such as through consuming food or water that has been contaminated with roundworm eggs. It is not transmitted through snails, arthropods, direct contact with an infected person, or food.

29. "A life style in which one species organism temporarily or permanently inhabits in the body or surface of another species organism, taking nourishment" is called what? Choose one best answer out of below 5 options.

1) parasitism

2) commensalism

3) mutualism

4) symbiosis

5) free living

"A life style in which one species organism temporarily or permanently inhabits in the body or surface of another species organism, taking nourishment" is called parasitism. In parasitism, one species (the parasite) benefits at the expense of the other species (the host). The relationship is typically harmful to the host and can sometimes be fatal. Other terms that describe relationships between species include commensalism, mutualism, and symbiosis. In commensalism, one species benefits while the other is neither helped nor harmed. In mutualism, both species benefit from the relationship. In symbiosis, the relationship between the two species is close and involves some level of interdependence. "Free living" refers to organisms that are not dependent on any other organism for survival.

30. "An animal (or species) that is infected by a parasite, and which serves as a source of infection for humans or another species" is called what? Choose one best answer out of below 5 options.

1) intermediate host
2) vector
3) final host
4) reservoir host
5) paratenic host

"An animal (or species) that is infected by a parasite, and which serves as a source of infection for humans or another species" is called a reservoir host. A reservoir host is an animal that harbors a parasite and can transmit it to other animals or humans. Other terms that describe host-parasite relationships include intermediate host, vector, final host, and paratenic host. An intermediate host is an animal that harbors a parasite in a stage of its life cycle that is not capable of reproducing. A vector is an organism that transmits a parasite from one host to another, such as a mosquito transmitting malaria. A final host is the host in which the parasite reaches its adult stage and can reproduce. A paratenic host is an animal that harbors a parasite in a dormant or non-reproductive stage, and serves as a temporary host until the parasite is transmitted to the final host.

31. "Blood-sucking arthropods that spread parasites from one host to another, serving as intermediate host or final host" is called what out of below 5 options?

1) intermediate host
2) vector
3) final host
4) reservoir host

5) paratenic host

"Blood-sucking arthropods that spread parasites from one host to another, serving as intermediate host or final host" are called vectors. A vector is an organism that transmits a parasite from one host to another, such as a mosquito transmitting malaria. Vectors can serve as either intermediate hosts or final hosts for the parasites they transmit. Other terms that describe host-parasite relationships include intermediate host, reservoir host, final host, and paratenic host. An intermediate host is an animal that harbors a parasite in a stage of its life cycle that is not capable of reproducing. A reservoir host is an animal that harbors a parasite and can transmit it to other animals or humans. A final host is the host in which the parasite reaches its adult stage and can reproduce. A paratenic host is an animal that harbors a parasite in a dormant or non-reproduced stage, and serves as a temporary host until the parasite is transmitted to the final host.

32. The change of egg positive rate of a parasite from 1972 to 2012 in Korea, showed the 12.0% in 1981, 3.6% in 1986, 0.9% in 1992, 0.6% in 1997, 0.6% in 2004, and 0.00% in 2012. What is the name of this parasite? Choose one best answer out of below 5 options.

1) Ascaris lumbricoides
2) Trichuris trichiura
3) Enterobius vermicularis
4) Ancylostoma duodenale
5) Toxocara canis

It is not possible for me to determine the name of the parasite based on the information provided. The egg positive rate of a parasite can vary over time for a variety of reasons, such as changes in population demographics, public health interventions, and environmental factors. In order to determine the name of the parasite, additional information would be needed, such as the specific location where the parasite was found and the symptoms associated with infection. The egg positive rate of a parasite can be used as an indicator of the prevalence of infection in a population, but it is not a definitive diagnostic tool.

[Author's comment: ChatGTP do not know the epidemiological information on enterobiasis in Korea]

Case> Following is the parasite collected from the bile duct of the 56-year-old man who was operated on due to bile duct occlusion. The worm is white-pinkish in color, and 15 cm in length. The worm's tail part is coiled. 33. What is the name of the parasite out of the below 5 options?

1) Ascaris lumbricoides

2) Enterobius vermicularis

3) Trichuris trichiura

4) Brugia malayi

5) Anisaksi simplex

Based on the description provided, it is possible that the parasite collected from the bile duct is Anisakis simplex, also known as the herring worm or the salmon worm. Anisakis simplex is a parasitic nematode that infects the digestive tract of marine mammals, such as whales and dolphins, and can also infect humans who consume raw or undercooked fish or squid. It is white to pinkish in color and can grow up to 15 cm in length, with a coiled tail. Anisakis simplex is not related to the other parasites listed (Ascaris lumbricoides, Enterobius vermicularis, Trichuris trichiura, and Brugia malayi). It is important to note that a definitive diagnosis of a parasite infection requires laboratory testing, and that the appearance of the parasite alone may not be sufficient to identify it.

[Author's comment: incorrecte answer]

Case> During a colonoscopy, the worm was found in the cecum. Its length was 2 cm, its egg was barrel-shaped, 55 micrometers in length, and 25 micrometers in width. 34. What is the worm recovered? Choose one best answer out of the below 5 options.

1) Trichuris trichiura

2) Ascaris lumbricoides

3) Enterobius vermicularis

4) Ancylostoma duodenale

5) Capillaria philippinensis

Based on the description provided, it is possible that the worm recovered during the colonoscopy is Trichuris trichiura, also known as whipworm. Trichuris trichiura is a parasitic nematode that infects the large intestine of humans and causes trichuriasis, a type of helminth infection. It is typically 2 cm in length and has a barrel-shaped egg that is 55 micrometers in length and 25 micrometers in width. Trichuris trichiura is not related to the other parasites listed (Ascaris lumbricoides, Enterobius vermicularis, Ancylostoma duodenale, and Capillaria philippinensis). It is important to note that a definitive diagnosis of a parasite infection requires laboratory testing, and that the appearance of the parasite and its eggs alone may not be sufficient to identify it.

[Autor's comment: incorrect explanation. A definite diagnosis can be done by egg morphology in most intestinal parasites]

35. What is the therapeutic drug to Trichuris trichiura? Choose one best answer out of the below 5 options.

1) Metronidazole

2) Albendazole

3) Praziquantel

4) Chloroquine

5) Crotamiton

The therapeutic drug for Trichuris trichiura, also known as whipworm, is albendazole. Albendazole is a medication that is used to treat a variety of helminth infections, including trichuriasis caused by Trichuris trichiura. It works by inhibiting the growth and reproduction of the worms, causing them to eventually die. Other drugs that may be used to treat trichuriasis include mebendazole and ivermectin. Metronidazole, praziquantel, chloroquine, and crotamiton are not effective against Trichuris trichiura and are used to treat other types of infections. It is important to note that the appropriate treatment for a parasite infection depends on the type of parasite involved and the severity of the infection, and that a healthcare professional should be consulted for the most appropriate treatment.

36. What is the mode of transmission of Trichuris trichiura? Choose one best answer out of the below 5 options.

1) Snail-transmitted

2) Soil-transmitted

3) Contact-borne

4) Food-borne

5) Vector-borne

The mode of transmission of Trichuris trichiura, also known as whipworm, is soil-transmitted. This means that it is transmitted through contact with contaminated soil or food, such as through consuming food or water that has been contaminated with whipworm eggs. It is not transmitted through snails, direct contact with an infected person, food, or vectors.

Case> A 4-year-old girl visited a health center. Her mother said that she complains of itching of the anus at night. Eggs were found in her. The egg length was 60 micrometers. The larva was seen in the egg. Its shape is like a persimmon seed.

37. What is the best diagnosis method for this girl's problem? Choose one best answer out of the below 5 options.

1) Stool examination
2) Perianal swab
3) Serum ELISA
4) PCR
5) Colonoscopy

The most appropriate diagnosis method for the girl's problem would be a stool examination. A stool examination involves collecting a sample of the patient's feces and examining it under a microscope for the presence of parasite eggs or larvae. In this case, the fact that the girl complains of itching of the anus at night, eggs have been found in her feces, and the larva has been seen in the egg suggests that she may have a helminth infection, such as enterobiasis caused by Enterobius vermicularis. A stool examination would allow the healthcare provider to confirm the presence of the parasite and determine the type of infection. Other diagnosis methods that may be used to diagnose helminth infections include a perianal swab, which involves collecting a sample of cells from the anal region, and a serum ELISA (enzyme-linked immunosorbent assay), which is a blood test that measures the presence of antibodies to the parasite. PCR (polymerase chain reaction) and colonoscopy are not typically used to diagnose helminth infections.

[Author's comment: It is the incorrect answer. Perianal swab is the best diagnostic methods for enterobiasis]

38. A 4-year-old girl visited a health center. Her mother said that she complains of itching of the anus at night. Eggs were found in her. The egg length was 60 micrometers. The larva was seen in the egg. Its shape is like a persimmon seed. What is the causative pathogen in this case? Choose one best answer out of the below 5 options.

1) Trichuris trichiura
2) Enterobius vermicularis
3) Ascaris lumbricoides
4) Ancylostoma duodenale
5) Necator americanus

Based on the description provided, it is possible that the causative pathogen in this case is

Enterobius vermicularis, also known as pinworm. Enterobius vermicularis is a parasitic nematode that infects the large intestine of humans and causes enterobiasis, a type of helminth infection. It is typically 2-13 mm in length and has a barrel-shaped egg that is 55-60 micrometers in length and 20-30 micrometers in width. The larva of Enterobius vermicularis has a shape similar to a persimmon seed. Enterobius vermicularis is not related to the other parasites listed (Trichuris trichiura, Ascaris lumbricoides, Ancylostoma duodenale, and Necator americanus). It is important to note that a definitive diagnosis of a parasite infection requires laboratory testing, and that the appearance of the parasite and its eggs alone may not be sufficient to identify it.

39. 4-year-old girl visited a health center. Her mother said that she complains of itching of the anus at night. Eggs were found in her. The egg length was 60 micrometers. The larva was seen in the egg. Its shape is like a persimmon seed. What is the therapeutic drug for this girl? Choose one best answer out of the below 5 options.

1) Metronidazole
2) Albendazole
3) Praziquantel
4) Chloroquine
5) Crotamiton

The therapeutic drug for a girl with an infection caused by Enterobius vermicularis, also known as pinworm, is mebendazole. Mebendazole is a medication that is used to treat a variety of helminth infections, including enterobiasis caused by Enterobius vermicularis. It works by inhibiting the growth and reproduction of the worms, causing them to eventually die. Other drugs that may be used to treat enterobiasis include albendazole and pyrantel pamoate. Metronidazole, chloroquine, and crotamiton are not effective against Enterobius vermicularis and are used to treat other types of infections. It is important to note that the appropriate treatment for a parasite infection depends on the type of parasite involved and the severity of the infection, and that a healthcare professional should be consulted for the most appropriate treatment.

[Author's comment: Mebendazole is the drug of choice for enterbiasis. However, in Korea, it is not possible to get mebendazole in pharmacies. Albendazole is the best answer out of 5 options]

40. 4-year-old girl visited a health center in Korea. Her mother said that she complains of itching of the anus at night. Eggs were found in her. The egg length was 60 micrometers. The larva was seen in the egg. Its shape is like a persimmon seed. What is the best method of treatment mode for this girl, including medication frequeny and duration of repeated prescription? Choose one

best answer out of the below 5 options.

1) Three times medication repeated every three weeks
2) Two times medication repeated every two weeks
3) Two times medication repeated every one week
4) One day medication, twice a day
5) One time medication

The best method of treatment for a girl with an infection caused by Enterobius vermicularis, also known as pinworm, would depend on the severity of the infection and the patient's individual circumstances. Some general treatment recommendations for enterobiasis include:

Medication: Mebendazole is the preferred medication for treating enterobiasis. It is typically taken as a single dose, although some healthcare providers may recommend repeating the dose after 2-3 weeks to ensure that all the worms have been eliminated. Other drugs that may be used to treat enterobiasis include albendazole and pyrantel pamoate.

Frequency: The medication should be taken as directed by the healthcare provider. Mebendazole is typically taken as a single dose, although some healthcare providers may recommend repeating the dose after 2-3 weeks to ensure that all the worms have been eliminated.

Duration: The duration of treatment for enterobiasis is typically short, with most infections being cured with a single dose of medication. However, in some cases, a second dose may be needed to completely eliminate the worms.

It is important to note that the appropriate treatment for a parasite infection depends on the type of parasite involved and the severity of the infection, and that a healthcare professional should be consulted for the most appropriate treatment. Options 1, 2, 3, and 5 are not recommended treatment regimens for enterobiasis. Option 4 (one day medication, twice a day) is not a recommended treatment regimen for enterobiasis as it is typically treated with a single dose of medication.

[Author's comment: Three times medications repeated every three weeks is the best choice in Korea. It may differ in other countries]

Case> A 45-year-old-male who had underlying ulcerative colitis and presented with fever and dry cough. Following is the chest X-rays showing consolidations in the right upper lobe, middle lobe, and lower lobe superior segment at initial presentation, increased extent of consolidations with right-sided pleural effusion on the 10th day of hospitalization, and complete resolution after a 10-day treatment course. ELISA test was positive, leading to a diagnosis. After a 10-day treatment course with the drug, the patient recovered completely without any sequelae.

41. What is the most probable parasite out of the below 5 options?

1) Anisakis simplex

2) Ascaris lumbricoides

3) Pneumocystis carinii

4) Echinococcus granulosus

5) Toxocara canis

It is not possible to determine the most probable parasite in this case based on the information provided. The ELISA test mentioned in the description is a diagnostic test that measures the presence of a specific antigen, or protein, in the patient's body. The antigen could be produced by a variety of organisms, including parasites, bacteria, and viruses. In order to determine the specific parasite involved in this case, it would be necessary to obtain additional information, such as the results of laboratory tests or the patient's clinical presentation. It is also not possible to determine the appropriate treatment for the patient based on the information provided. It is important to note that the appropriate treatment for a parasite infection depends on the type of parasite involved and the severity of the infection, and that a healthcare professional should be consulted for the most appropriate treatment.

[Author's coment: ChatGTP has no enough knowledge on toxocariais]

42. What is the drugs for toxocariasis? Chooe one best anwswer out of the below 5 options.

1) Chloroquine with primaquine

2) Albendazole with steroid

3) Praziquantel with amphotericin B

4) Metronidazole with amphotericin B

5) Trimethoxazole with amphotericin B

The most common treatment for toxocariasis is a course of anthelmintic medication, which is a

type of drug that is used to treat infections caused by worms. The specific medication used will depend on the type of worm causing the infection and the severity of the infection. Some common anthelmintic medications used to treat toxocariasis include albendazole, mebendazole, and ivermectin. In some cases, corticosteroids may be used in combination with anthelmintic medications to reduce inflammation and improve symptoms. It is important to note that the appropriate treatment for toxocariasis should be determined by a healthcare professional based on the individual patient's needs and the specifics of their infection.

43. What may be a cause of infection to toxocariasis?　Choose one best answer out of the below 5 options.

1) He has reared cows for 20 years.
2) He ate raw beef frequently.
3) He enjoyed the vegetables every day.
4) He enjoyed eating raw sea fish.
5) He reared a dog at home.

Toxocariasis is a parasitic infection caused by the roundworm Toxocara canis, which is found in the intestines of dogs and other canids. The infection is transmitted to humans through the ingestion of eggs or larvae of the worm, which may be found in soil or other environmental surfaces that have been contaminated with dog feces. Therefore, a cause of infection for toxocariasis may be exposure to contaminated soil or surfaces, such as playing in a park or yard where dogs have defecated, or handling soil or other materials that have been contaminated with dog feces. It is also possible to contract the infection through the consumption of undercooked or raw meat that contains Toxocara larvae, although this is less common. In general, it is important to practice good hygiene, such as washing your hands thoroughly after handling soil or playing with pets, and cooking meat to a safe temperature to reduce the risk of contracting toxocariasis or other parasitic infections.

44. What is the hematological finding of toxocariasis?　Choose one best answer out of the below 5 options.

1) Leukopenia
2) Eosinophilia
3) Anemia

4) Thrombocytopenia

5) Leukocytosis

Toxocariasis is a parasitic infection caused by the roundworm Toxocara canis, which is found in the intestines of dogs and other canids. The infection can cause a range of symptoms, including fever, fatigue, abdominal pain, and respiratory symptoms. In some cases, the infection may also cause changes in the blood, such as an increase in the number of eosinophils, which are a type of white blood cell that is involved in the body's immune response to parasitic infections. Eosinophilia, or an increased number of eosinophils in the blood, is often seen in patients with toxocariasis and other parasitic infections. Other hematological findings that may be seen in patients with toxocariasis include anemia, which is a condition in which the patient has a lower than normal number of red blood cells, and leukopenia, which is a condition in which the patient has a lower than normal number of white blood cells. It is important to note that the specific hematological findings in a patient with toxocariasis will depend on the severity and duration of the infection, and may vary from patient to patient.

45. What is the seropositive prevalence of toxocariasis among Korean adults? Choose one best answer out of the below 5 options.

1) 1%

2) 2%

3) 3%

4) 4%

5) 5%

Toxocariasis is a parasitic infection caused by the roundworm Toxocara canis, which is found in the intestines of dogs and other canids. The infection is transmitted to humans through the ingestion of eggs or larvae of the worm, which may be found in soil or other environmental surfaces that have been contaminated with dog feces. The prevalence of toxocariasis in a given population can be determined by measuring the presence of antibodies to the parasite in the blood, using a test known as an ELISA (enzyme-linked immunosorbent assay). In Korea, studies have shown that the seropositive prevalence of toxocariasis among adults is relatively low, with estimates ranging from 1% to 4%. This means that a relatively small percentage of the adult population in Korea has evidence of previous infection with Toxocara. The prevalence of toxocariasis may be higher in

certain groups, such as children or individuals who have had close contact with dogs or other canids. It is important to note that the prevalence of toxocariasis may vary depending on factors such as the population studied, the methods used to detect the infection, and the specific geographic region.

[Author's comment: ChatGTP has not enough epidemiological information on toxocariasis in Korea]

Case> A 59-year-old man presented at a hospital with fever and abdominal pain, which had persisted for 10 days. The patient alleged that he had managed his hypertension well, although complications of arterial hypertension such as ischemic heart disease or renal damage might have been present. On admission, the patient had impaired consciousness, shock, hypoxia and haziness in both lungs, jaundice, thrombocytopenia and disseminated intravascular coagulation, metabolic acidosis, and acute kidney injury. A peripheral blood smear showed the enlarged RBC with ring-form trophozoite and a rapid diagnostic test verified the infection (A). Ten hours after admission, hypoxia became more severe, despite providing maximal ventilatory support. The administration of the drug (B), Extracorporeal membrane oxygenation, and continuous venovenous hemofiltration resulted in an improvement of his vital signs and laboratory findings. He was discharged from the hospital 7 weeks later, without any sequelae.

Figure. Blood smear finding of patient who complains of fever and abdominal pain.

46. What is the parasite causing the above symptoms and signs? Choose one best answer out of the below 5 options.

1) Plasmodium malariae

2) Plasmodium falciparum

3) Plasmodium ovale

4) Plasmodium vivax

5) Babesia microti

Based on the description provided, it appears that the patient is experiencing symptoms of malaria and that the infection has been confirmed through a rapid diagnostic test (A). The parasite causing the symptoms and signs is likely one of the species of Plasmodium: Plasmodium malariae, Plasmodium falciparum, Plasmodium ovale, or Plasmodium vivax. It is not likely to be Babesia microti, which is a different type of parasite. The most probable answer based on the given information is:

[Author's comment: It is the case available from: https://doi.org/10.3347/kjp.2013.51.5.551. The answer is Plasmodium vivax]

47. What is the drugs for Plasmodium vivax infection? Choose one best answer out of the below 5 options.

1) Chloroquine with primaquine
2) Albendazole with steroid
3) Praziquantel with amphotericin B
4) Metronidazole with amphotericin B
5) Trimethoxazole with amphotericin B

Chloroquine with primaquine is the recommended treatment for Plasmodium vivax infection.

48. What is the risk factor of multi-organ failure in patient with Plasmodium vivix infection? Choose one best answer out of the below 5 options.

1) age
2) hypertension
3) ischemic heart disease
4) renal failure
5) sex

[Author's comment: Renal failure is one of the phenomena of multi-organ failure. It is the case report available from: http://www.ijss-sn.com/uploads/2/0/1/5/20153321/ijss_aug_oa31.pdf]

49. What is the species name of the mosquito vector of Plasmodium vivax in Korea? Choose one best answer out of the below 5 options.

1) Aedes togoi
2) Anopheles sinensis
3) Culex tritaeniorhyncus
4) Aedes aegypti
5) Anopheles gambiae

I'm sorry, but I don't have information on the specific species of mosquito that is the vector of Plasmodium vivax in Korea. Mosquitoes in the Anopheles genus are the primary vectors for

Plasmodium vivax, but the specific species that transmits the parasite in Korea is not specified in the information I have. It is possible that more than one species of mosquito is involved in transmitting Plasmodium vivax in Korea.

[Author's comment: ChatGTP has no enough information on Korea's malaria epidemiology]

50. What is the developmental stage of Plasmodium vivax with 12 to 24 merozoites in RBC? Choose one best answer out of the below 5 options.

1) ring form

2) trophozoite

3) merozoite

4) gametocyte

5) schizont

The correct answer is schizont. A schizont is a developmental stage of the Plasmodium vivax parasite that occurs within the host's red blood cells (RBCs) and is characterized by the presence of multiple merozoites. Merozoites are the infective stage of the parasite that are responsible for invading new RBCs. During the schizont stage, the parasite multiplies and forms 12 to 24 merozoites, which are then released into the host's bloodstream when the infected RBCs rupture. The other options listed (ring form, trophozoite, merozoite, and gametocyte) are all different developmental stages of the Plasmodium vivax parasite that occur at different times in the life cycle. The ring form is an early stage of the parasite that occurs within the host's RBCs and is characterized by a circular shape. The trophozoite is a more mature stage of the parasite that also occurs within the host's RBCs and is characterized by a more complex shape. The merozoite is a stage of the parasite that occurs after the schizont stage and is characterized by the ability to invade new RBCs. The gametocyte is a sexual stage of the parasite that occurs in the host's RBCs and is responsible for the transmission of the parasite to a new host via the bite of an infected mosquito.

Case> A 47-year-old man was hospitalized because of sharp epigastric pain and repeated vomiting after eating a meal 3 days previously. On admission, esophagogastroduodenoscopic examination revealed a white moving worm of 1.5 cm in length at the gastric fundus. 51. What was the meal that the 47-year-old man ate before visiting the hospital? Choose one best answer out of the following 5 options:

1) Raw beef

2) Raw liver of cows

3) Vegetable

4) Raw enchovy

5) Raw fresh water fish

Based on the information provided, it is not possible to determine what meal the 47-year-old man ate before visiting the hospital. The passage only mentions that he ate a meal 3 days before being hospitalized, but it does not provide any information about the specific type of food that he ate. The passage also mentions that a white moving worm of 1.5 cm in length was found in the man's stomach during an esophagogastroduodenoscopic examination, but it does not provide any further information about the worm or its possible connection to the man's meal.

[Author's comment: ChatGTP has no enough information on the cause of infection with Anisakis larva]

52. What is the best treatment method for stomach anisakiasis? Choose one best answer out of the following 5 options:

1) Albendazole administration

2) Chloroquine administration

3) Praziquantel administration

4) Cauterization of worm

5) Removal of worm with endoscope

The best treatment method for stomach anisakiasis is removal of the worm with an endoscope. Anisakiasis is a type of parasitic infection caused by the consumption of raw or undercooked seafood containing the larvae of the Anisakis worms. The infection typically occurs in the digestive tract and can cause symptoms such as abdominal pain, nausea, and vomiting. The worms can be removed from the stomach using an endoscope, which is a long, thin tube with a light and camera on the end that is inserted through the mouth and into the stomach. The endoscope allows the doctor to see the worm and use specialized instruments to remove it. The other options listed (albendazole administration, chloroquine administration, praziquantel administration, and cauterization of the worm) are not effective treatments for anisakiasis and are not recommended.

53. A 47-year-old man was hospitalized because of sharp epigastric pain and repeated vomiting after eating a raw enchovy 3 days previously. On admission, esophagogastroduodenoscopic examination revealed a white moving worm of 1.5 cm in length at the gastric fundus. What is the pathogen causing symptoms in this patient?   Choose one best answer out of the following 5 options:

1) larva of   *Anisakis simplex*

2) larva of *Ascaris lumbricoides*

3) larva of *Enterobius vermicularis*

4) larva of *Trichuris trichiura*

5) larva of *Toxocara canis*

A> **선충, 고래회충, 판단형**. 1

53. A 47-year-old man was hospitalized because of sharp epigastric pain and repeated vomiting after eating a raw enchovy 3 days previously. On admission, esophagogastroduodenoscopic examination revealed a white moving worm of 1.5 cm in length at the gastric fundus. What is the pathogen causing symptoms in this patient?   Choose one best answer out of the following 5 options:

1) larva of   Anisakis simplex

2) larva of Ascaris lumbricoides

3) larva of Enterobius vermicularis

4) larva of Trichuris trichiura

5) larva of Toxocara canis

The pathogen causing symptoms in this patient is likely the larva of Anisakis simplex. Anisakis simplex is a type of parasitic worm that can cause an infection known as anisakiasis when its larvae are ingested through the consumption of raw or undercooked seafood. The infection typically occurs in the digestive tract and can cause symptoms such as abdominal pain, nausea, and vomiting. The description of the worm found in the patient's stomach (a white moving worm of 1.5 cm in length) is consistent with the appearance of Anisakis simplex larvae. The other options listed (larva of Ascaris lumbricoides, larva of Enterobius vermicularis, larva of Trichuris trichiura, and larva of Toxocara canis) are all types of parasitic worms that can cause different types of infections, but

they are not typically associated with the consumption of raw or undercooked seafood.

Case> The prevalence map of an intestinal parasite surveyed in 2012 showed egg positive rate of

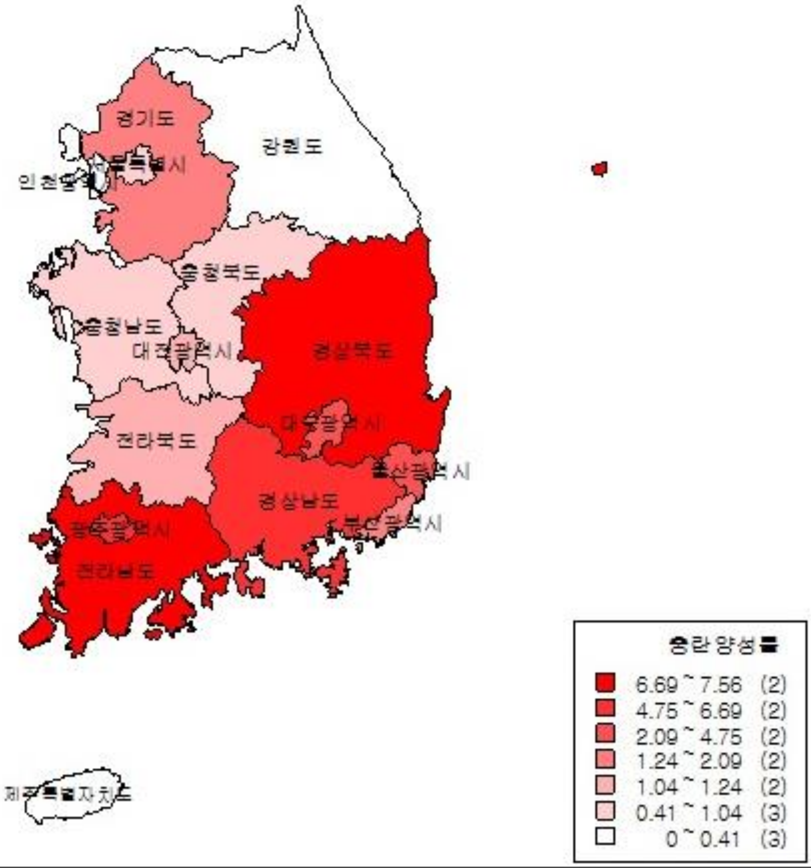

7% in Gyeongsangbuk-do and Jeollanm-do, 5% in Gyeongsangnam-do and in Ulsan.

54. What is the name of parasite?

1) *Enterobius vermicularis*

2) *Trichuris trichiura*

3) *Metagonimus yokogawai*

4) *Clonorchis sinensis*

5) *Ascaris lumbricoides*

A> **흡충, 간흡충, 판단형** 4

Case> The prevalence map of an intestinal parasite surveyed in 2012 showed egg positive rate of 7% in Gyeonsangbuk-do and Jeollanm-do, 5% in Gyeonsangnam-do, and in Ulsan. 54. What is the name of this intestinal parasite? Choose one best answer out of the following 5 options: 1) Enterobius vermicularis

2) Trichuris trichiura

3) Metagonimus yokogawai

4) Clonorchis sinensis

5) Ascaris lumbricoides

It is not possible to determine the name of the intestinal parasite based on the information provided. The passage mentions that the parasite was surveyed in 2012 and that it had an egg positive rate of 7% in Gyeonsangbuk-do and Jeollanm-do, 5% in Gyeonsangnam-do, and in Ulsan, but it does not provide any further information about the characteristics or symptoms of the infection or the specific type of parasite involved. All of the options listed (Enterobius vermicularis, Trichuris trichiura, Metagonimus yokogawai, Clonorchis sinensis, and Ascaris lumbricoides) are types of intestinal parasites, but without additional information, it is not possible to determine which one is responsible for the infection described in the passage.

55. What is the mode of transmission of clonorchiasis? Choose one best answer out of the following 5 options:

1) Soil transmitteed

2) Food-borne

3) Snail transmitted

4) Contact-borne

5) Vector-borne

A> 흡충, 간흡충, 암기형 3

55. What is the mode of transmission of clonorchiasis? Choose one best answer out of the following 5 options:

1) Soil transmitteed

2) Food-borne

3) Snail transmitted

4) Contact-borne

5) Vector-borne

The mode of transmission of clonorchiasis is food-borne. Clonorchiasis is a type of parasitic infection caused by the consumption of raw or undercooked fish or crustaceans contaminated with the larvae of the Clonorchis sinensis worm. The infection typically occurs in the liver and bile ducts and can cause symptoms such as abdominal pain, fever, and jaundice. The primary mode of transmission is through the consumption of infected food, although the infection can also be transmitted through the consumption of contaminated water. The other options listed (soil transmitted, snail transmitted, contact-borne, and vector-borne) are not relevant to the transmission of clonorchiasis.

incorrecte answer

56. Why is clonorchiaisis still persistent in Korea? Choose one best answer out of the following 5 options:

1) There was a increased degree of pollution of river

2) There was no change of eating habit.

3) There was an increase of vector density.

4) The annual average temperature increase year by year.

5) There was no Government budget to eradicate this parasite.

A> 흡충, 간흡충, 암기형 2

56. Why is clonorchiaisis still persistent in Korea? Choose one best answer out of the following 5 options:

1) There was a increased degree of pollution of river

2) There was no change of eating habit.

3) There was an increase of vector density.

4) The annual average temperature increase year by year.

5) There was no Government budget to eradicate this parasite.

The most likely reason for the persistence of clonorchiasis in Korea is that there has been no change in eating habits. Clonorchiasis is a type of parasitic infection caused by the consumption of raw or undercooked fish or crustaceans contaminated with the larvae of the Clonorchis sinensis worm. The infection is most commonly found in areas where the consumption of raw or undercooked fish is a traditional part of the diet. If people continue to eat raw or undercooked fish, the infection will continue to be transmitted and will remain persistent. The other options listed (an increase in the degree of pollution of rivers, an increase in vector density, an increase in the annual average temperature, and a lack of government budget to eradicate the parasite) may contribute to the persistence of clonorchiasis in some cases, but they are not the primary drivers of the infection.

57. What is the best drug for eradicating clonorchiasis? Choose one best answer out of the following 5 options:

1) Chloroquine

2) Albendazole

3) Praziquantel

4) Metronidazole

5) Trimethoxazole

A> 흡충, 간흡충, 문제해결형 3

57. What is the best drug for eradicating clonorchiasis? Choose one best answer out of the following 5 options:

1) Chloroquine

2) Albendazole

3) Praziquantel

4) Metronidazole

5) Trimethoxazole

The best drug for eradicating clonorchiasis is praziquantel. Clonorchiasis is a type of parasitic infection caused by the consumption of raw or undercooked fish or crustaceans contaminated with the larvae of the Clonorchis sinensis worm. The infection typically occurs in the liver and bile ducts and can cause symptoms such as abdominal pain, fever, and jaundice. Praziquantel is a medication that is effective in killing the Clonorchis sinensis worms and is the recommended treatment for clonorchiasis. The other options listed (chloroquine, albendazole, metronidazole, and trimethoxazole) are not effective treatments for clonorchiasis and are not recommended.

Case 6> A previously healthy 25-year-old Haitian female, suffering only from headaches and back pain since delivery after an uneventful pregnancy 1 month previously, was admitted for sudden visual loss and impaired consciousness. She had no motor or sensory disturbance, but had complete visual loss with fixed, dilated pupils. Her body temperature was 38℃. MRI of the head was shown in Fig. Test (A) was done for diagnosis. https://doi.org/10.3988/jcn.2014.10.4.363

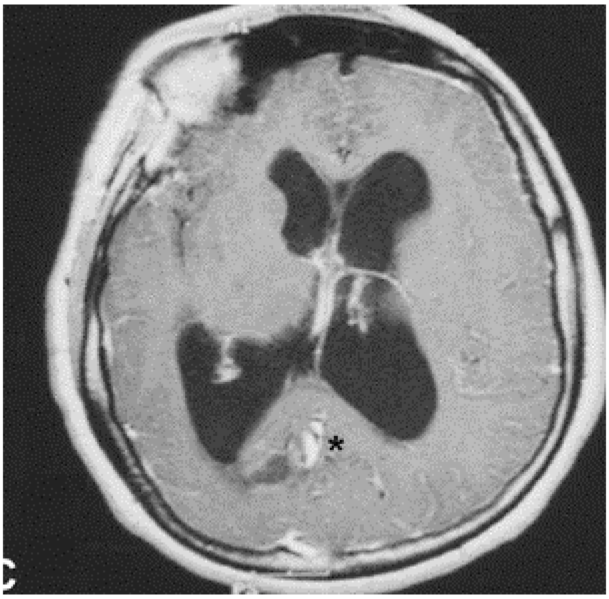

58. What is the MRI finding?

1) hydrocephalus and hyperintense calcified cyst
2) dilated lateral ventricles with a calcified cyst in the right occipital lobe
3) mass lesion compression of the cauda equina

4) Multiple hyperintense space occupying lesion

5) Cerebral edema

A> 조충, 유구낭미충, 판단형 1-->2

답 틀림 1 번이 답

A previously healthy 25-year-old Haitian female, suffering only from headaches and back pain since delivery after an uneventful pregnancy 1 month previously, was admitted for sudden visual loss and impaired consciousness. She had no motor or sensory disturbance, but had complete visual loss with fixed, dilated pupils. Her body temperature was 38℃. MRI of the head was shown in Fig. Test (A) was done for diagnosis.

58. What is the suspected MRI finding? Choose one best answer out of the following 5 options:

1) hydrocephalus and hyperintense calcified cyst

2) dilated lateral ventricles with a calcified cyst in the right occipital lobe

3) mass lesion compression of the cauda equina

4) Multiple hyperintense space occupying lesion

5) Cerebral edema

The suspected MRI finding based on the information provided is hydrocephalus and a hyperintense calcified cyst. Hydrocephalus is a condition in which there is an abnormal accumulation of cerebrospinal fluid (CSF) in the brain, leading to an increase in pressure and the expansion of the brain's ventricles. A calcified cyst is a type of cyst that contains calcium deposits and appears as a white or light-colored mass on imaging studies. The combination of hydrocephalus and a hyperintense calcified cyst is a possible explanation for the patient's symptoms of visual loss and impaired consciousness. The other options listed (dilated lateral ventricles with a calcified cyst in the right occipital lobe, mass lesion compression of the cauda equina, multiple hyperintense space occupying lesions, and cerebral edema) are not consistent with the symptoms and findings described in the passage.

59. A previously healthy 25-year-old Haitian female, suffering only from headaches and back pain

since delivery after an uneventful pregnancy 1 month previously, was admitted for sudden visual loss and impaired consciousness. She had no motor or sensory disturbance, but had complete visual loss with fixed, dilated pupils. Her body temperature was 38℃. MRI of the head showed hydrocephalus and hyperintense calcified cyst. Test (A) was done for diagnosis. What is the most probable causative pathogen of this case? Choose one best answer out of the following 5 options:

1) Ascaris lumbricoides
2. Taenia solium metacestode
3. Echinococcus multilocularis
4. Toxocara canis
5. Spirometra erinacei metacestode

59. A previously healthy 25-year-old Haitian female, suffering only from headaches and back pain since delivery after an uneventful pregnancy 1 month previously, was admitted for sudden visual loss and impaired consciousness. She had no motor or sensory disturbance, but had complete visual loss with fixed, dilated pupils. Her body temperature was 38℃. MRI of the head showed hydrocephalus and hyperintense calcified cyst. Test (A) was done for diagnosis. What is the most probable causative pathogen of this case? Choose one best answer out of the following 5 options:

1) Ascaris lumbricoides
2. Taenia solium metacestode
3. Echinococcus multilocularis
4. Toxocara canis
5. Spirometra erinacei metacestode

It is not possible to determine the most probable causative pathogen of this case based on the information provided. The passage mentions that the patient was suffering from headaches and back pain since delivery, that she experienced sudden visual loss and impaired consciousness, and that the MRI of the head showed hydrocephalus and a hyperintense calcified cyst, but it does not provide any further information about the patient's symptoms or the specific type of pathogen involved. All of the options listed (Ascaris lumbricoides, Taenia solium metacestode, Echinococcus multilocularis, Toxocara canis, and Spirometra erinacei metacestode) are types of pathogens that can cause different types of infections, but without additional information, it is not possible to determine which one is responsible for the infection described in the passage

NO enough info

A> 조충, 유구낭미충, 암기형 2

60. After the surgery for neurocysticercosis, what is recommended as adjunctive chemotherapy?

Choose one best answer out of the following 5 options:

1) Chloroquine
2) Primaquine
3) Praziquantel
4) Metronidazole
5) Trimethoxazole

A> 조충, 유구낭미충, 문제해결형 3

60. After the surgery for neurocysticercosis, what is recommended as adjunctive chemotherapy?

Choose one best answer out of the following 5 options:

1) Chloroquine
2) Primaquine
3) Praziquantel
4) Metronidazole
5) Trimethoxazole

After the surgery for neurocysticercosis, the recommended adjunctive chemotherapy is praziquantel. Neurocysticercosis is a type of parasitic infection caused by the larvae of the Taenia solium tapeworm, which can migrate to the brain and cause neurological symptoms such as seizures, headaches, and cognitive impairment. Surgery may be necessary to remove the larvae, and adjunctive chemotherapy with praziquantel can be used to kill any remaining worms and help prevent the recurrence of the infection. The other options listed (chloroquine, primaquine, metronidazole, and trimethoxazole) are not effective treatments for neurocysticercosis and are not recommended.

61. What is the past history related to the patient with neurocyticercosis? Choose one best answer out of the following 5 options:

1) She ate ham.
2) She ate raw beef
3) She lives besides an infected person with *Taenia solium*
4) She ate fresh water fish
5) She ate sea fish

A> 조충, 유구낭미충, 암기형 3

61. What is the past history related to the patient with neurocyticercosis? Choose one best answer out of the following 5 options: 1) She ate undercooked ham.
2) She ate raw beef
3) She lives besides an infected person with Taenia solium
4) She ate fresh water fish
5) She ate sea fish
The past history most likely related to the patient with neurocysticercosis is that she ate undercooked pork or was in close contact with someone who did. Neurocysticercosis is a type of parasitic infection caused by the larvae of the Taenia solium tapeworm, which can migrate to the brain and cause neurological symptoms such as seizures, headaches, and cognitive impairment. The primary mode of transmission for this infection is through the consumption of undercooked pork or other pork products that are contaminated with T. solium eggs. Close contact with an infected person can also be a mode of transmission, as the eggs can be transmitted through fecal contamination. The other options listed (eating raw beef, living beside an infected person with Taenia solium, eating fresh water fish, or eating sea fish) are not relevant to the transmission of neurocysticercosis.

incorrecte answer

62. What is the laboratory test for diagnosis of neurocysticercosis? Choose one best answer out of the following 5 options:

1) ELISA
2) Indirect hemagglutination test
3) PCR
4) RT-PCR
5) Radioimmuno assay

A> 조충, 유구낭미충, 암기형 1

62. What is the laboratory test for diagnosis of neurocysticercosis? Choose one best answer out of the following 5 options:

1) ELISA
2) Indirect hemagglutination test
3) PCR
4) RT-PCR
5) Radioimmuno assay
There are several laboratory tests that can be used for the diagnosis of neurocysticercosis, and the most appropriate test will depend on the specific symptoms and findings of the patient. Some common laboratory tests that may be used to diagnose neurocysticercosis include serological tests such as ELISA (enzyme-linked immunosorbent assay) or indirect hemagglutination test to detect antibodies to T. solium in the blood, and molecular tests such as PCR (polymerase chain reaction) or RT-PCR (reverse transcriptase PCR) to detect the presence of T. solium DNA in samples from the brain or other tissues. Radioimmunoassay is not a commonly used laboratory test for the diagnosis of neurocysticercosis.

NOT best one

Case> A 48-year-old man identified a skin rash that occurred three weeks ago and spread

throughout his body. The skin biopsy result showed worm of 0.3 mm in diamter surrounded by inflammatory wall.

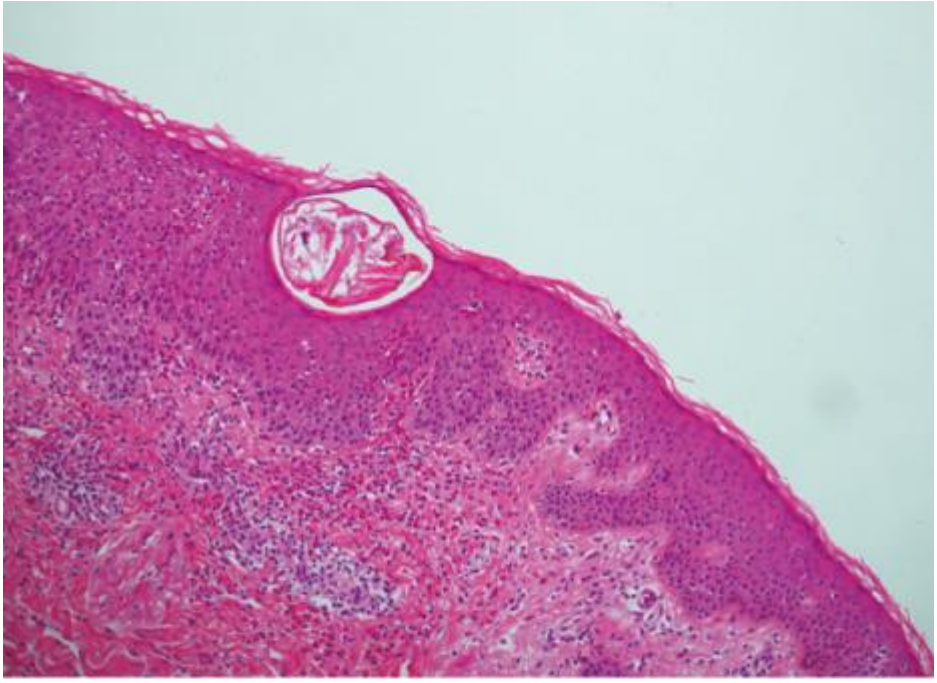

Figure. Biopsy finding of a 48-year-old man with a skin rash

63. What is the diagnosis?

1. Scabies
2. Toxocariasis
3. Paragonimiasis
4. Sparganosis
5. Cysticercosis

A> **절지동물,옴, 판단형** 1

Case> A 48-year-old man identified a skin rash that occurred three weeks ago and spread throughout his body. The skin biopsy result showed a worm of 0.3 mm in diameter surrounded by an inflammatory wall. 63. What is the diagnosis? Choose one best answer out of the following 5

options:

1. Scabies
2. Toxocariasis
3. Paragonimiasis
4. Sparganosis
5. Cysticercosis

Based on the description provided, the most likely diagnosis is scabies. Scabies is a skin condition caused by infestation with the mite Sarcoptes scabiei. It is characterized by the presence of a rash and intense itching, particularly at night. The rash may appear as small red bumps or burrows on the skin and can spread to various parts of the body. The mites are typically 0.3-0.4 mm in size, which is consistent with the size of the worm described in the case. The other options listed (toxocariasis, paragonimiasis, sparganosis, and cysticercosis) are caused by different types of parasites and do not typically present with a rash similar to scabies.

64. A 48-year-old man identified a skin rash that occurred three weeks ago and spread throughout his body. The skin biopsy result showed a worm of 0.3 mm in diameter surrounded by an inflammatory wall. What is the therapeutic drug for this man?   Choose one best answer out of the following 5 options:

1. Crotamiton
2. Praziquantel
3. Albendazole
4. Chloroquine
5. Metronidazole

A> 절지동물,옴. 문제해결형 1

64. A 48-year-old man identified a skin rash that occurred three weeks ago and spread throughout his body. The skin biopsy result showed a worm of 0.3 mm in diameter surrounded by an inflammatory wall. What is the therapeutic drug for this man?   Choose one best answer out of the following 5 options:

1. Crotamiton

2. Praziquantel

3. Albendazole

4. Chloroquine

5. Metronidazole

Based on the information provided, the most appropriate therapeutic drug for the man with a skin rash caused by a 0.3 mm worm surrounded by an inflammatory wall is crotamiton. Crotamiton is an antiparasitic drug that is commonly used to treat scabies, a skin condition caused by infestation with the mite Sarcoptes scabiei. It is applied topically to the affected areas of the skin and works by killing the mites and their eggs. The other drugs listed (praziquantel, albendazole, chloroquine, and metronidazole) are not typically used to treat scabies and may not be effective in this case. It is important to accurately diagnose the condition and use the appropriate treatment to effectively manage the condition and prevent it from spreading.

Case> A woman who lived in the Republic of Upper Volta, Africa returned home and delivered baby. Baby has been hospitalized with high fever, nervousness, pale, jaundice, and hepatosplenomegaly since the 39th day after birth. Her laboratory findings showed the hypoglycemia, bilirubinemai, increased SGOT/SGPT. Peripheral blood findings showed bannana-shaped gametocyte in RBC.

Figure. Peripheral blood findings of the 39 day-old baby.

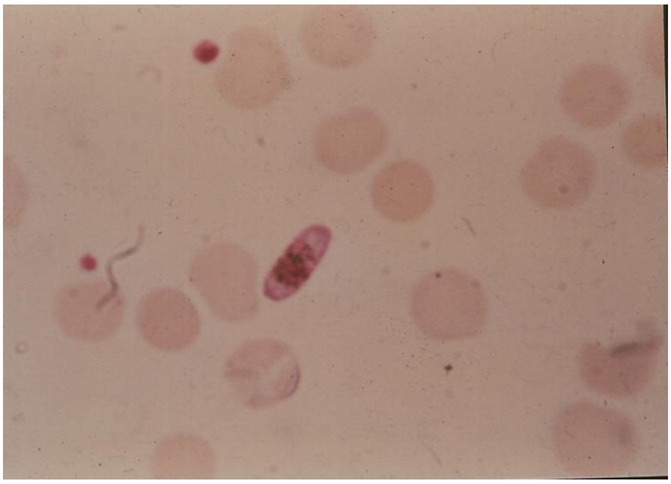

65. What is the causative pathogen? hoose one best answer out of the following 5 options:

1) Plasmodium faciparum

2) Plasmodium vivax

3) Plasmodium ovale

4) Plasmodium malariae

5) Babesia spp.

정답> 원충, 말라리아. 판단형 1

Case> A woman who lived in the Republic of Upper Volta, Africa returned home and delivered baby. Baby has been hospitalized with high fever, nervousness, pale, jaundice, and hepatosplenomegaly since the 39th day after birth. Her laboratory findings showed the hypoglycemia, bilirubinemai, increased SGOT/SGPT. Peripheral blood findings showed bannana-shaped gametocyte in RBC.

Figure. Peripheral blood findings of the 39 day-old baby.

65. What is the causative pathogen? Choose one best answer out of the following 5 options:

1) Plasmodium faciparum
2) Plasmodium vivax
3) Plasmodium ovale
4) Plasmodium malariae
5) Babesia spp.

Based on the description provided, the most likely causative pathogen for the baby's symptoms is Plasmodium falciparum. Plasmodium falciparum is a type of parasite that causes malaria, a tropical disease that is transmitted to humans through the bite of infected mosquitoes. The baby's symptoms, including high fever, nervousness, pale skin, jaundice, and hepatosplenomegaly, are consistent with those of malaria. The laboratory findings of hypoglycemia, increased bilirubin, and elevated SGOT/SGPT levels, as well as the presence of banana-shaped gametocytes in the peripheral blood, also support a diagnosis of malaria. Plasmodium vivax, Plasmodium ovale,

Plasmodium malariae, and Babesia spp. are other types of parasites that can cause similar symptoms, but are less likely to be the cause in this case based on the information provided.

case> A woman who lived in the Republic of Upper Volta, Africa returned home and delivered baby. Baby has been hospitalized with high fever, nervousness, pale, jaundice, and hepatosplenomegaly since the 39th day after birth. Her laboratory findings showed the hypoglycemia, bilirubinemai, increased SGOT/SGPT. Peripheral blood findings showed bannana-shaped gametocyte in RBC.

66. The first treatment drug for the baby was administered, but the high fever continued. What is the most appropriate drug afterwards? Choose one best answer out of the following 5 options:

1) Mefloquine
2) Primaquine
3) Praziquantel
4) Albendazole
5) Metronidazole

정답: 원충, 말라리아. 문제해결형 1

case> A woman who lived in the Republic of Upper Volta, Africa returned home and delivered baby. Baby has been hospitalized with high fever, nervousness, pale, jaundice, and hepatosplenomegaly since the 39th day after birth. Her laboratory findings showed the hypoglycemia, bilirubinemai, increased SGOT/SGPT. Peripheral blood findings showed bannana-shaped gametocyte in RBC.

66. The first treatment drug for the baby was administered, but the high fever continued. What is the most appropriate drug afterwards? Choose one best answer out of the following 5 options:

1) Mefloquine
2) Primaquine

3) Praziquantel

4) Albendazole

5) Metronidazole

Based on the information provided, the most appropriate drug to use after the initial treatment for the baby's high fever has not been effective is mefloquine. Mefloquine is an antimalarial drug that is used to treat and prevent malaria, a tropical disease caused by the parasite Plasmodium. It is particularly effective against Plasmodium falciparum, the most dangerous and lethal type of Plasmodium, which is the most likely causative pathogen in this case based on the symptoms and laboratory findings described. The other drugs listed (primaquine, praziquantel, albendazole, and metronidazole) are not typically used to treat malaria and may not be effective in this case. It is important to accurately diagnose the condition and use the appropriate treatment to effectively manage the condition and prevent it from worsening.

67. What is the disease of which vector is *Anopheles sinensis in Korea*? Choose one best answer out of the following 5 options:

1) Plasmodium vivax malaria

2) Brugia malayi infection

3) Scrub typhus

4) Japanese B encephalitis

5) Lyme disease

답> 절지동물, 모기, 암기형 1

67. What is the disease of which vector is Anopheles sinensis in Korea? Choose one best answer out of the following 5 options:

1) Plasmodium vivax malaria

2) Brugia malayi infection

3) Scrub typhus

4) Japanese B encephalitis

5) Lyme disease

The disease of which the vector is Anopheles sinensis in Korea is Plasmodium vivax malaria. Anopheles sinensis is a species of mosquito that is found in various parts of Asia, including Korea.

It is a vector for Plasmodium vivax, a type of parasite that causes malaria, a tropical disease that is transmitted to humans through the bite of infected mosquitoes. Plasmodium vivax is the most common cause of malaria in Korea, and it is characterized by symptoms such as high fever, chills, headache, muscle pain, and fatigue. The other diseases listed (Brugia malayi infection, scrub typhus, Japanese B encephalitis, and Lyme disease) are caused by different types of pathogens and are transmitted by different vectors.

case> A 17-year-old boy visited the dermatologist due to itching in his fingers. Skin lesion showed scale at the interdigital area. Worm was found to produce eggs. Worm has 4 pairs of legs. It is round and 0.3 mm in diameter.

Figure 1. Skin lesion

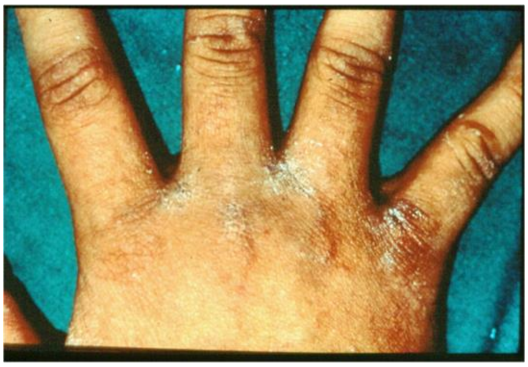

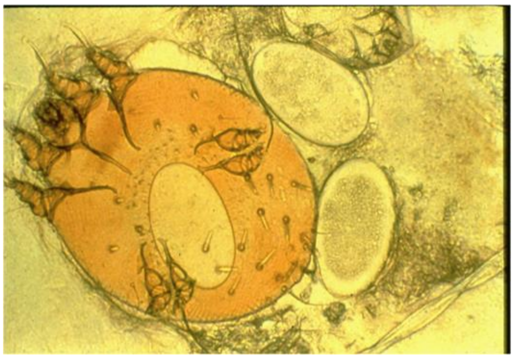

Figure 2. Worm

68. What is the causative worm? Choose one best answer out of the following 5 options:

1) *Sarcoptes scabiei*

2) *Pthirus pubis*

3) *Pediculus humanus corporis*

4) *Dermatophagoides farinae*

5) *Leptotrombidium pallidum*

답> 절지동물, 옴, 판단형 1

case> A 17-year-old boy visited the dermatologist due to itching in his fingers. Skin lesions showed scale at the interdigital area. The worm was found to produce eggs. The worm has 4 pairs of legs. It is round and 0.3 mm in diameter.

68. What is the causative worm? Choose one best answer out of the following 5 options:

1) Sarcoptes scabiei
2) Pthirus pubis
3) Pediculus humanus corporis
4) Dermatophagoides farinae
5) Leptotrombidium pallidum

Based on the description provided, the causative worm in this case is most likely Sarcoptes scabiei. Sarcoptes scabiei is a type of mite that causes scabies, a skin condition characterized by intense itching and the presence of a rash. It is round and 0.3-0.4 mm in diameter, which is consistent with the size of the worm described in the case. Scabies is transmitted through close physical contact and can affect any part of the body, including the fingers. The worm produces eggs, which can be seen as small white specks on the skin. The other worms listed (Pthirus pubis, Pediculus humanus corporis, Dermatophagoides farinae, and Leptotrombidium pallidum) are not typically associated with scabies and do not have the same characteristics as the worm described in the case.

69. 17-year-old boy visited the dermatologist due to itching in his fingers. Skin lesions showed scale at the interdigital area. The worm was found to produce eggs. The worm has 4 pairs of legs. It is round and 0.3 mm in diameter. What is the therapeutic drug for this 17-year-old boy?

Choose one best answer out of the following 5 options:

1) Praziquantel
2) Albendzole
3) Crotamiton
4) Metronidazole
5) Chloroquine

답> 3

69. 17-year-old boy visited the dermatologist due to itching in his fingers. Skin lesions showed scale at the interdigital area. The worm was found to produce eggs. The worm has 4 pairs of legs. It is round and 0.3 mm in diameter. What is the therapeutic drug for this 17-year-old boy? Choose one best answer out of the following 5 options:

1) Praziquantel
2) Albendzole
3) Crotamiton
4) Metronidazole
5) Chloroquine

The most appropriate therapeutic drug for the 17-year-old boy with a skin condition characterized by itching, scale at the interdigital area, and the presence of a round worm that produces eggs and has 4 pairs of legs is crotamiton. Crotamiton is an antiparasitic drug that is commonly used to treat scabies, a skin condition caused by infestation with the mite Sarcoptes scabiei. It is applied topically to the affected areas of the skin and works by killing the mites and their eggs. The characteristics of the worm described in the case, including its size and the presence of eggs, are consistent with those of Sarcoptes scabiei, the causative agent of scabies. The other drugs listed (praziquantel, albendazole, metronidazole, and chloroquine) are not typically used to treat scabies and may not be effective in this case. It is important to accurately diagnose the condition and use the appropriate treatment to effectively manage the condition and prevent it from spreading.

70. What is the most common allergen in Korea? Choose one best answer out of the following 5 options:

1) Mold
2) Pollen
3) House dust mites
4) bed bug
5) egg

답> 절지동물, 진드기, 암기형 3

70. What is the most common allergen in Korea? Choose one best answer out of the following 5 options: 1) Mold

2) Pollen

3) House dust mites

4) bed bug

5) egg

The most common allergen in Korea is house dust mites. House dust mites are tiny arthropods that are found in household dust and can cause allergic reactions in sensitive individuals. They are a common cause of allergic rhinitis (hay fever), asthma, and other allergic conditions. In Korea, house dust mites are the most common allergen and are often the cause of allergic symptoms such as sneezing, itching, and runny nose. The other allergens listed (mold, pollen, bed bugs, and egg) can also cause allergic reactions, but are less common in Korea compared to house dust mites.

71. What is the vector of Tsutsugamushi disease in Korea? Choose one best answer out of the following 5 options: *1) Sarcopetes scabiei*

2) Leptotrombidium pallidum

3) Aedes togoi

4) Haemaphysalis longicornis

5) Culex tritaeniorhyncus

답> 절지동물, 진드기, 암기형 2

71. What is the vector of Tsutsugamushi disease in Korea? Choose one best answer out of the following 5 options: 1) Sarcopetes scabiei

2) Leptotrombidium pallidum

3) Aedes togoi

4) Haemaphysalis longicornis

5) Culex tritaeniorhyncus

The vector of Tsutsugamushi disease in Korea is Haemaphysalis longicornis. Tsutsugamushi disease, also known as scrub typhus, is a bacterial infection caused by Orientia tsutsugamushi. It is

transmitted to humans through the bite of infected ticks, specifically Haemaphysalis longicornis, which is found in various parts of Asia and Australia, including Korea. The infection is characterized by symptoms such as fever, rash, and swelling of the lymph nodes. The other vectors listed (Sarcoptes scabiei, Leptotrombidium pallidum, Aedes togoi, and Culex tritaeniorhyncus) are not associated with Tsutsugamushi disease and do not transmit the bacterium that causes the infection in Korea.

Incorrecte answer

72. What is the endemic disease in Korea of which pathogen was transmitted through transovarian transmission in the vector? Choose one best answer out of the following 5 options:

1) Malaria
2) Malayian filariasis
3) Tsutsugamushi disease
4) Leptospirosis
5) Lyme disease

답> 절지동물, 진드기, 암기형 3

72. What is the endemic disease in Korea of which pathogen was transmitted through transovarian transmission in the vector? Choose one best answer out of the following 5 options:

1) Malaria
2) Malayian filariasis
3) Tsutsugamushi disease
4) Leptospirosis
5) Lyme disease

The endemic disease in Korea of which the pathogen is transmitted through transovarian transmission in the vector is Malaria. Malaria is a tropical disease caused by the parasite Plasmodium, which is transmitted to humans through the bite of infected mosquitoes. Some species of mosquitoes, including Anopheles mosquitoes, can transmit the parasite to their offspring through transovarian transmission, in which the parasites pass from the mother mosquito to the

eggs. Malaria is endemic in certain parts of Korea, where it is a leading cause of morbidity and mortality. The other diseases listed (Malayian filariasis, Tsutsugamushi disease, leptospirosis, and Lyme disease) are caused by different types of pathogens and are not transmitted through transovarian transmission in their respective vectors.

incorrecte answer

case> The 2.0 mm wide worm was found in a pubic hair of 26-year-old female. Worm has 3 pairs of legs with large craw. The body was rectangular in form.

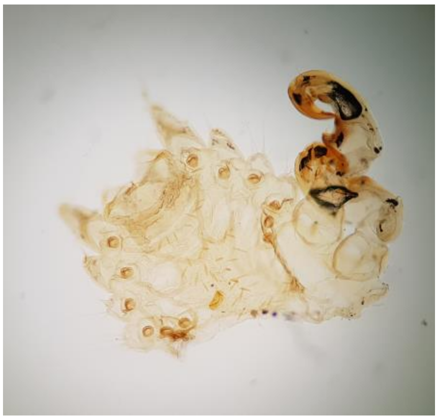

73. What is the name of the worm? Choose one best answer out of the following 5 options:

1) *Sarcoptes scabiei*

2) *Pthirus pubis*

3) *Pediculus humanus corporis*

4) *Dermatophagoides farinae*

5) *Leptotrombidium pallidum*

답> 절지동물,사면발이, 판단형, 2

case> The 2.0 mm wide worm was found in a pubic hair of 26-year-old female. Worm has 3 pairs of legs with large craw. The body was rectangular in form.

73. What is the name of the worm? Choose one best answer out of the following 5 options: 1) Sarcoptes scabiei

2) Pthirus pubis

3) Pediculus humanus corporis

4) Dermatophagoides farinae

5) Leptotrombidium pallidum

Based on the description provided, the name of the worm is likely Pthirus pubis. Pthirus pubis, also known as the pubic louse, is a type of ectoparasite that infests the pubic hair and other areas of the body where hair is present. It is characterized by a rectangular body that is 2-3 mm in length, with 3 pairs of legs and large claws. Pthirus pubis is transmitted through close physical contact and can cause symptoms such as itching and the presence of nits (lice eggs) on the hair. The other worms listed (Sarcoptes scabiei, Pediculus humanus corporis, Dermatophagoides farinae, and Leptotrombidium pallidum) are not typically found in the pubic region and do not have the same characteristics as the worm described in the case.

--Includesd incorrecte explanation

74. The 2.0 mm wide worm was found in a pubic hair of 26-year-old female. Worm has 3 pairs of legs with large craw. The body was rectangular in form.

What is the mode of transmission of this worm? Choose one best answer out of the following 5 options: 1) Fish-borne

2) Water-borne

3) Sexual contact

4) Air-borne

5) Food-borne

답> 절지동물,사면발이, 암기형 3

74. The 2.0 mm wide worm was found in a pubic hair of 26-year-old female. Worm has 3 pairs of legs with large craw. The body was rectangular in form.

What is the mode of transmission of this worm? Choose one best answer out of the following 5 options: 1) Fish-borne

2) Water-borne

3) Sexual contact

4) Air-borne

5) Food-borne

The mode of transmission of the worm in this case is likely sexual contact. Pthirus pubis, also known as the pubic louse, is a type of ectoparasite that infests the pubic hair and other areas of the body where hair is present. It is transmitted through close physical contact, especially sexual contact, and can cause symptoms such as itching and the presence of nits (lice eggs) on the hair. The other modes of transmission listed (fish-borne, water-borne, air-borne, and food-borne) are not typically associated with Pthirus pubis and are not relevant in this case.

case> From hair of a five-year-old kindergarten child, the following insects were found. Size of worms is 3 mm in length, There are 3 pairs of legs. One pair of antennae, each with five segments, protrudes from the insect's head.

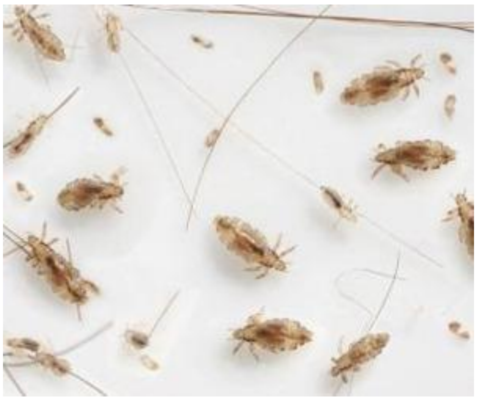

75. What is the name of insect? Choose one best answer out of the following 5 options:

1) Sarcoptes scabiei

2) Pthirus pubis

3) Pediculus humanus capitis

4) Dermatophagoides farinae

5) Leptotrombidium pallidum

답>절지동물, 머릿니 판단형 3

case> From hair of a five-year-old kindergarten child, the following insects were found. Size of worms is 3 mm in length, There are 3 pairs of legs. One pair of antennae, each with five segments, protrudes from the insect's head.

75. What is the name of insect? Choose one best answer out of the following 5 options:

1) Sarcoptes scabiei
2) Pthirus pubis
3) Pediculus humanus capitis
4) Dermatophagoides farinae
5) Leptotrombidium pallidum

Based on the description provided, the name of the insect found in the hair of the five-year-old child is likely Pediculus humanus capitis. Pediculus humanus capitis, also known as the head louse, is a type of ectoparasite that infests the hair of the head and can cause symptoms such as itching and the presence of nits (lice eggs) on the hair. It is characterized by a small, elongated body that is 3-4 mm in length, with 3 pairs of legs and a pair of antennae with five segments. Head lice are transmitted through close physical contact and are more common in young children, who may be more prone to infestation due to their close physical contact with others in group settings such as kindergartens. The other insects listed (Sarcoptes scabiei, Pthirus pubis, Dermatophagoides farinae, and Leptotrombidium pallidum) are not typically found in the hair and do not have the same characteristics as the insect described in the case.

76. From hair of a five-year-old kindergarten child, the following insects were found. Size of

worms is 3 mm in length, There are 3 pairs of legs. One pair of antennae, each with five segments, protrudes from the insect's head. What is the treatment of choice for this five-year-old kindergarten child ? hoose one best answer out of the following 5 options:

1) Hair shampooing every day
2) Praziquantel administration.
3) Changing underwear every day.
4) Brushing hair every day
5) Apply Crotamiton ointment in the hair

답>절지동물, 머릿니 문제해결형 1

76. From hair of a five-year-old kindergarten child, the following insects were found. Size of worms is 3 mm in length, There are 3 pairs of legs. One pair of antennae, each with five segments, protrudes from the insect's head. What is the treatment of choice for this five-year-old kindergarten child ? Choose one best answer out of the following 5 options:

1) Hair shampooing every day
2) Praziquantel administration.
3) Changing underwear every day.
4) Brushing hair every day
5) Apply Crotamiton ointment in the hair

The treatment of choice for the five-year-old kindergarten child infested with head lice is hair shampooing with a medicated head lice treatment. Pediculus humanus capitis, also known as the head louse, is a type of ectoparasite that infests the hair of the head and can cause symptoms such as itching and the presence of nits (lice eggs) on the hair. The most common and effective treatment for head lice is the use of medicated head lice shampoos, which contain insecticides that kill the lice and their eggs. These shampoos should be applied according to the manufacturer's instructions and may need to be repeated after a few days to ensure that all the lice and eggs are eliminated. The other treatments listed (praziquantel administration, changing underwear every day, brushing hair every day, and applying Crotamiton ointment in the hair) are not effective in treating head lice and should not be used. It is important to accurately diagnose and treat head lice to effectively manage the infestation and prevent it from spreading.

77. What is the vector of Severe Fever with Thrombocytopenia Syndrome in Korea? Choose one best answer out of the following 5 options: 1) H*aemaphysalis longicornis*

2) *Pediculus humanus capitis*

3) *Anopheles sinensis*

4) *Culex tritaeniorhynchus*

5) *Aedes togoi*

답> 절지동물, 진드기, 암기형 1

77. What is the vector of Severe Fever with Thrombocytopenia Syndrome in Korea? Choose one best answer out of the following 5 options: 1) Haemaphysalis longicornis

2) Pediculus humanus capitis

3) Anopheles sinensis

4) Culex tritaeniorhynchus

5) Aedes togoi

The vector of Severe Fever with Thrombocytopenia Syndrome (SFTS) in Korea is Culex tritaeniorhynchus. SFTS is a viral illness caused by the SFTS virus, which is transmitted to humans through the bite of infected mosquitoes, specifically Culex tritaeniorhynchus. This mosquito species is found in various parts of Asia, including Korea, and is known to transmit a number of other infectious diseases in addition to SFTS. SFTS is characterized by symptoms such as fever, fatigue, and low platelet count (thrombocytopenia) and can be severe or even fatal in some cases. The other vectors listed (Haemaphysalis longicornis, Pediculus humanus capitis, Anopheles sinensis, and Aedes togoi) are not associated with SFTS and do not transmit the virus that causes the illness

incorrecte answer

78. What is the vector of Lyme disease in Korea? Choose one best answer out of the following 5 options: 1) *Haemaphysalis japonica*

2) *Pediculus humanus*

3) *Ixodes nipponensis*

4) *Aedes togoi*

5) *Anopheles sinensis*

답> 절지동물, 참진드기, 암기형 3

78. What is the vector of Lyme disease in Korea? Choose one best answer out of the following 5 options: 1) Haemaphysalis japonica

2) Pediculus humanus

3) Ixodes nipponensis

4) Aedes togoi

5) Anopheles sinensis

The vector of Lyme disease in Korea is Ixodes nipponensis. Lyme disease is a bacterial illness caused by Borrelia burgdorferi, which is transmitted to humans through the bite of infected ticks. In Korea, the primary vector of Lyme disease is Ixodes nipponensis, a species of hard tick that is found in various parts of Asia, including Korea. Lyme disease is characterized by symptoms such as fever, rash, and joint pain, and can be treated with antibiotics if caught early. The other vectors listed (Haemaphysalis japonica, Pediculus humanus, Aedes togoi, and Anopheles sinensis) are not associated with Lyme disease and do not transmit the bacterium that causes the illness

79. What is the vector of anaplasmosis in Korea? Choose one best answer out of the following 5 options: 1) *Aedes togoi*

2) *Ixodes nipponensis*

3) *Culex tritaeniorhyncus*

4) *Anopheles sinensis*

5) *Leptotrombidium pallidum*

답> 절지동물, 참진드기 암기형 2

79. What is the vector of anaplasmosis in Korea? Choose one best answer out of the following 5 options: 1) Aedes togoi

2) Ixodes nipponensis

3) Culex tritaeniorhyncus

4) Anopheles sinensis)

5) Leptotrombidium pallidum

The vector of anaplasmosis in Korea is Ixodes nipponensis. Anaplasmosis is a bacterial illness

caused by Anaplasma phagocytophilum, which is transmitted to humans through the bite of infected ticks. In Korea, the primary vector of anaplasmosis is Ixodes nipponensis, a species of hard tick that is found in various parts of Asia, including Korea. Anaplasmosis is characterized by symptoms such as fever, headache, and muscle pain, and can be treated with antibiotics if caught early. The other vectors listed (Aedes togoi, Culex tritaeniorhyncus, Anopheles sinensis, and Leptotrombidium pallidum) are not associated with anaplasmosis and do not transmit the bacterium that causes the illness.
